# Microfluidics and organ-on-a-chip technologies: A systematic review of the methods used to mimic bone marrow

**DOI:** 10.1371/journal.pone.0243840

**Published:** 2020-12-11

**Authors:** Gabriel Santos Rosalem, Libardo Andrés Gonzáles Torres, Estevam Barbosa de Las Casas, Fernando Augusto Siqueira Mathias, Jeronimo Conceição Ruiz, Maria Gabriela Reis Carvalho

**Affiliations:** 1 Mechanical Engineering Graduate Program, Universidade Federal de Minas Gerais, Belo Horizonte, Brazil; 2 Institute of Science and Technology, Universidade Federal dos Vales do Jequitinhonha e Mucuri, Diamantina, Brazil; 3 Department of Structural Engineering, Universidade Federal de Minas Gerais, Belo Horizonte, Brazil; 4 Biosystems and Genomics Group, René Rachou Institute, Oswaldo Cruz Foundation, Belo Horizonte, Brazil; 5 Graduate Program in Computational and Systems Biology of the Institute Oswaldo Cruz (PGBCS/IOC/Fiocruz), Rio de Janeiro, Brazil; CNR NANOTEC - Istituto di Nanotecnologia del CNR, ITALY

## Abstract

Bone marrow (BM) is an organ responsible for crucial processes in living organs, e. g., hematopoiesis. In recent years, Organ-on-a-Chip (OoC) devices have been used to satisfy the need for *in vitro* systems that better mimic the phenomena occurring in the BM microenvironment. Given the growing interest in these systems and the diversity of developed devices, an integrative systematic literature review is required. We have performed this review, following the PRISMA method aiming to identify the main characteristics and assess the effectiveness of the devices that were developed to represent the BM. A search was performed in the Scopus, PubMed, Web of Science and Science Direct databases using the keywords ((“bone marrow” OR “hematopoietic stem cells” OR “haematopoietic stem cells”) AND (“organ in a” OR “lab on a chip” OR “microfluidic” OR “microfluidic*” OR (“bioreactor” AND “microfluidic*”))). Original research articles published between 2009 and 2020 were included in the review, giving a total of 21 papers. The analysis of these papers showed that their main purpose was to study BM cells biology, mimic BM niches, model pathological BM, and run drug assays. Regarding the fabrication protocols, we have observed that polydimethylsiloxane (PDMS) material and soft lithography method were the most commonly used. To reproduce the microenvironment of BM, most devices used the type I collagen and alginate. Peristaltic and syringe pumps were mostly used for device perfusion. Regarding the advantages compared to conventional methods, there were identified three groups of OoC devices: perfused 3D BM; co-cultured 3D BM; and perfused co-cultured 3D BM. Cellular behavior and mimicking their processes and responses were the mostly commonly studied parameters. The results have demonstrated the effectiveness of OoC devices for research purposes compared to conventional cell cultures. Furthermore, the devices have a wide range of applicability and the potential to be explored.

## Introduction

BM is responsible for producing blood, bone, and immune system cells in a process known as hematopoiesis [1]. BM is located in trabecular cavities of bones and comprises several cell types, such as hematopoietic stem cells (HSCs), mesenchymal stem cells (MSCs), osteoblasts, and other niche cells [2], and also the heterogeneous extracellular matrix (ECM) and an intricate network of blood vessels. ECM of BM provides a mechanical microstructure and a suitable biochemical framework for growth, differentiation, and maintenance of cell functions [3]. The microvascular system of BM is formed by arterial and sinusoidal vessels [4] that perform essential functions such as the delivery of nutrients, cell traffic, and waste removal [5].

BM is composed of three regions called niches: endosteal, central, and perivascular niche. Niches are local tissue microenvironments that maintain and regulate stem cells and are characterized by different physical properties, proteins, and cell types [6]. The endosteal niche contains type I and IV collagen, osteopontin, and fibronectin; it is home for osteoblasts and osteoclasts and its typical stiffness ranges from 35 to 40 kPa [7–9]. The central niche is the least stiff region of BM, with stiffness of approximately 0.3 kPa. It contains laminin, fibronectin, heparin, hyaluronic acid, adipocytes, macrophages, and fibroblasts [10–12]. The perivascular region, in turn, contains type IV collagen, fibronectin, and laminin. The cells that inhabit this niche include endothelial, stromal cells, and MSCs; the stiffness of this tissue varies from 2 to 10 kPa [8, 13, 14].

Another important characteristic of the niches is the distribution of oxygen in the BM microenvironment. The oxygen concentration controls essential cellular processes for the maintenance of the physiological behavior of tissues and organs and is considered a key parameter in the regulation of the survival and quiescence of HSCs in the BM niches [15]. The oxygen concentration is not distributed evenly in the microenvironment of the niches: it is lower in the endosteal niche and gradually increases towards the perivascular niche [16] because of proximity of blood vessels that provide oxygen supply.

Understanding physiological and pathological processes associated with BM and its response to different therapeutic strategies is a complex task that requires studying the influence of physical, mechanical, biochemical, and biological characteristics of the cellular microenvironment on the behavior of cells and tissues [17].

The behavior of cells and the influence of different stimuli on living cells and tissues have been attracting the attention of researchers for more than a century already. One of the first approaches that the scientists used in this field were two-dimensional cell cultures [18] and animal models [19]. Though widely used, the 2D cell culture system gives a poor representation of cellular microenvironment, and the resulting *in vitro* experiments do not reflect the phenomena occurring *in vivo* [20]. While giving a better representation of cell microenvironment in the human body than the 2D cell cultures, animal models also have some drawbacks, including genetic differences between animals and humans, differences in the cell microenvironment compared to humans, and ethical issues related to animal experiments [21]. An alternative to these approaches that emerged in the last decades is the use of cultures in three-dimensional (3D) environments. These systems allow growing cells in a more realistic environment than their 2D counterpart, hence improving the phenotypic representations cell functions and providing a possibility of reducing the use of animal testing [22]. However, even with the great advancement of 3D culture models, they still cannot mimic some fundamental features of organs, e. g., tissue-tissue interactions, biochemical gradients, and mechanical forces acting in the cellular microenvironment [23]. These limitations directly affect the obtained results that tend to be oversimplified. The oversimplified results, in turn, hinder the understanding of disease evolution in humans and the development of more effective therapeutic strategies [24].

Under these circumstances, devices called Organ-on-a-chip (OoC) provide a promising alternative for studying cellular behavior in a more realistic environment. OoC can be defined as microengineered biomimetic systems [25] designed to study physiological organ- or tissue-specific cell behavior in microfluidic chambers [26]. Microfluidic devices are miniaturized systems, with channels ranging from 10 to 1000 micrometers in size, allowing the flow of small (between 10^−9^ and 10^−18^ liters) amounts of fluid [27]. These devices can mimic the micro-architecture, cell environment, signaling and mechanical, physical, and biochemical characteristics of a living organ [28]. One of the important advantages of these systems is that they take into account the fluid flow intrinsic to living organisms.

The production of OoC involves specific micro- and nano- fabrication techniques, including replica modeling [20], microcontact printing [29], soft lithography [30, 31], 3D printing [32] and injection molding [33, 34] and the selection of suitable materials for each application. Examples of these materials are polydimethylsiloxane (PDMS), polycarbonate (PC), polymethylmethacrylate (PMMA), polystyrene (PS), polyimide (PI), and polyvinyl chloride (PVC) [35].

Numerous advantages have been reported for the use of OoC, including the flexibility of device design, high level of control of the system parameters, diversity of experimental applications, few required cells, possibility to handle a single cell, real-time analysis using microscopy, possibility to use a coupled analysis system, availability of systems for controlled co-cultures with fluid perfusion, and the reduction of reagent consumption [36]. Some biological advantages include greater accuracy in simulating drug delivery using biomimetic 3D structures [37], the possibility of studying interactions between different cells by controlling factors (nutrients and growth factors) and ECM properties in OoC [38], and control of mechanical signaling induced by tissue deformation and physiological flow [17, 26]. Compared to conventional models, OoC allows a better representation of cell and tissue characteristics not only for fundamental biological research but also for clinical diagnostics and treatment [39].

Currently, there are available several studies in the literature focusing on the development of devices mimicking different organs and tissues (lung [40], kidney [41], gut [42], heart [43], blood vessels [44], spleen [45], liver [46], and brain [47]). There is also a growing interest in mimicking BM: several OoC devices representing physiological and pathophysiological conditions and assessing therapeutic strategies for common diseases like leukemia have been developed over the last years.

Considering the critical role of the BM system in the states of health or disease, its biological complexity, diverse functions in the human body, and pathologies that affect it, a growing scientific interest in mimicry of BM’s functions and niches using OoC devices, and a wide range of available materials and production methods for OoCs, the present review aims to identify the state-of-the-art of OoC devices for BM representation. This paper seeks to address the following guideline questions: *Which are the main purposes and objectives of using OoC to mimic BM in biological research*? *Which fabrication processes and materials are used for the devices*? *Which mechanisms are used for the perfusion*? *Which materials are used to mimic the BM ECM*? *Which cell types are commonly used in studies mimicking the BM microenvironment*? *Which methods and processes are used to evaluate the efficiency of OoC devices mimicking BM*? And *Are the existing devices effective*?

## Methods

To achieve the objective of the study, we have performed an integrative systematic review following the PRISMA–Preferred Reporting Items for Systematic Reviews and Meta-Analyses–method [48, 49]. The first step in the preparation of the review was outlining the guiding questions about the subject. Then, the search strategy (the choice of databases) and the eligibility criteria (inclusion and exclusion criteria) were defined. The final step was to retrieve and summarize the data, results, and conclusions from the selected studies.

Articles published in English between January 1st, 2009 and July 24th, 2020, were included in the review. We chose Scopus, PubMed, Web of Science, and ScienceDirect databases, as they cover the fields of engineering, physics, chemistry, and biology, and searched for combinations of the following key expressions in the title, abstract, or keywords of the articles: (("bone marrow" OR "hematopoietic stem cells" OR "haematopoietic stem cells") AND ("organ on a chip" OR "lab on a chip" OR "microfluidics" OR "microfluidic*" OR (“bioreactor” AND “microfluidic*"))).

Only original research publications that had an abstract and presented experimental studies on BM mimicking with microfluidic technology (e.g., organ-on-a-chip and lab-on-a-chip) and the applications of these studies, reported qualitative and quantitative results, and referred to human or animal cells were included. Publications with only computational approaches, as well as reviews were excluded. A three-step analysis protocol was followed in the screening process. In the first step, we screened the title and abstract of the publications. Publications that did not contain the words "microfluidic" or "chip" combined with "marrow", "hematopoietic", and "haematopoietic" were excluded in the first round (as works “not containing a microfluidic/OoC approach”). After that, the publications that did not report results or did not refer to BM were also excluded (as works with “irrelevant outcomes”). In the last step, the introduction, results, discussion, and conclusions of the selected publications were systematically screened. Publications that did not mimic at least one BM feature (e.g., specific niches, cell niche interactions, or BM microenvironment) or that assessed only isolated BM phenomena, without associating them with the structural aspects of the BM (e. g., hematopoietic stem cells differentiation in a planar substrate), were also excluded (as works with “poor mimicking”). After that, all remaining publications were selected for qualitative analysis. For the purpose of the analysis, we created a matrix with information related to the most relevant aspects of OoC devices, including physical and biological characteristics, materials and fabrication processes, main phenomena studied and analyses used to validate the efficiency of OoC technology.

## Results and discussion

**Fig 1:**
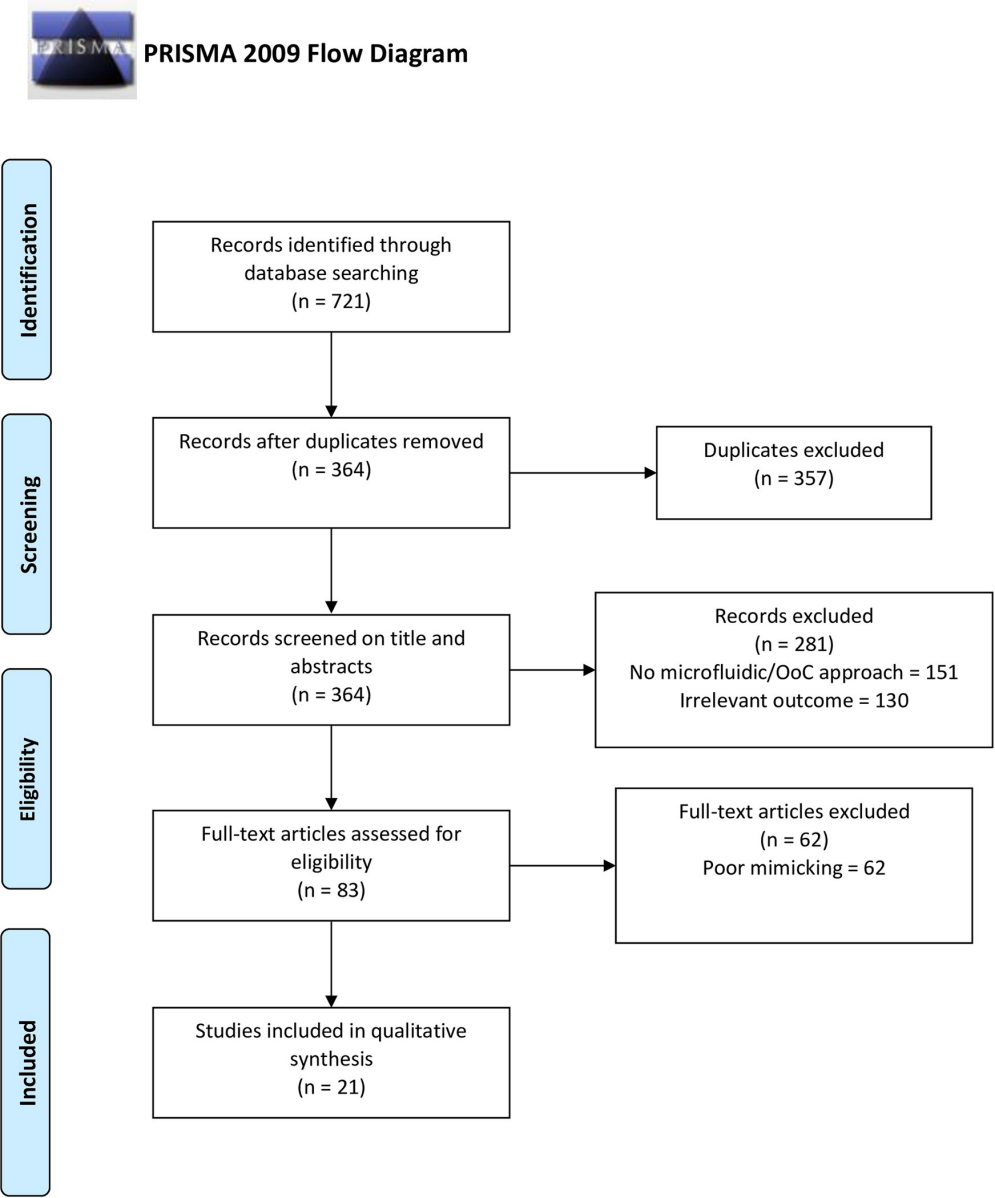
PRISMA flow diagram. Systematic review of bone marrow mimicking methods.

The number of articles retrieved from all databases was 721, of which, 272 were obtained from Scopus, 218 from PubMed, 205 from Web of Science, and 179 from ScienceDirect database. Before applying the exclusion criteria, we identified and removed duplicate publications (n = 357). Then, the publications classified as ‘not containing microfluidic/OoC approach’ and having ‘irrelevant outcomes’ were excluded (n = 281). After that, we screened the introduction, results, discussion, and conclusions sections of the articles and excluded the publications classified as ‘poor mimicking’ (n = 62). The detailed list of all excluded publications and the reasons for exclusion are given in S1 Appendix in Supporting Information section. After all screening procedures, the remaining 21 articles were included in this systematic review (Fig 1).

We have organized the data to highlight the results that are most relevant to the state-of-the-art of the investigated question and have classified the publications from the perspective of two main aspects: the device’s features (application of the device, research goals, materials used, fabrication methods, fluidic mechanisms, microenvironment materials, and cell types) and the device performance (methods of assessment and control and improvements that the device offers compared to other research methods). The publications are chronologically organized in Table 1.

**Table 1:** General parameters and improvements provided by the microfluidic approach to BM mimicking.

Reference	Device application/Research goals	Device material/Fabrication method	Fluidic mechanism	Cells type/Microenvironment material	Control/Evaluation methods	Main advantages and improvements provided by the device
Sung et al., Lab Chip 2009 [50]	*Modeling pathological BM*^*b*^	Silicon/Conventional photo-lithography	Peristaltic pump	h-abnormal myeloblastic cell (AML)/Alginate	Static 2D culture/Methods: Cell viability; Cytotoxicity assay	*Perfused 3D hydrogel matrix*
	*Drug assay*^*c*^					Provides a more realistic microenvironment compared to conventional culture, improving the understanding of the metabolism-dependent toxicity of an anti-cancer drug
	Mimicking the pharmacokinetic profile of a drug in the human body					
Carrion et al., Biotechnol Bioeng 2010 [51]	*BMC biology*^*a*^*/BM niche mimicking*^*d*^	PDMS/Soft-lithography	Reservoir	h-UVEC; h-fibroblast; h-MSC/Fibrinogen	No control culture/Methods: Analysis of vasculogenesis; Cell migration; Cell adhesion	*Co-culture in 3D hydrogel matrix*
						Provides a microenvironment able to improve the vasculogenesis and successfully shows the perivascular behavior of MSCs *ex vivo*
	Studying the molecular regulation of perivascular stem cell niches in a 3D device					
Sung et al., Lab chip 2010 [52]	*Modeling pathological BM*	PDMS/Soft-lithography	Gravity-induced flow	h-abnormal myeloblastic cell (AML)/Alginate	Static 2D culture/Methods: Cell viability; Cytotoxicity assay	*Perfused 3D hydrogel matrix*
	*Drug assay*					Provides a more realistic microenvironment, improving the understanding of the metabolism-dependent toxicity of an anti-cancer drug compared to conventional culture
	Mimicking the pharmacokinetic-pharmacodynamic profile of a drug in multi-organs	Silicone/Engineered layer				
Zhang et al., Tissue Eng–Part C Methods 2014 [53]	*Modeling pathological BM/BM niche mimicking*	PDMS/Soft-lithography	Syringe pump	h-OSB; h-plasma cell myeloma (MM)/OSB-generated	No control culture/Methods: Cell proliferation; Cell-tissue interactions	*Co-culture in perfused 3D OSB-generated matrix*
						Provides a microenvironment based on the perfused culture of MM in ECM OSB-generated capable of improving the mimic of MM-endosteal niche interactions, assisting in the development of novel therapeutics to abrogate microenvironment-driven drug resistance
	Development of a microfluidic 3D system for preserving the bone marrow-MMC interactions					
Khin et al., Cancer Res 2014 [54]	*Modeling pathological BM*	Plastic^e^/Injection molding	Reservoir	h-plasma cell myeloma (MM); h-MSC/Type I collagen	Static 3D culture/Methods: Pharmacodynamic assay; Chemotherapeutic assay; Cell viability	*Co-culture in 3D hydrogel matrix*
	*Drug assay*					Provides a microenvironment that improves the understanding of the dynamics of interactions between tumor and stroma in response to therapeutic agents *in vitro*
	Studying drug response in MM through an interdisciplinary platform that mimics the 3D microenvironment of BM					
Torisawa et al., Nat Methods 2014 [55]	*BMC biology*	PDMS/Soft-lithography	Syringe pump	a-BMC/Type I collagen	No control culture/Methods: Histological analysis; Cytokines assay; Cell viability	*Co-culture in perfused 3D hydrogel/bone powder matrix*
	*Drug assay*			Demineralized bone powder		
	*Cultivating living marrow with a functional hematopoietic niche in vitro*					*Provides a biomimetic microphysiological system improve the prediction of the in vivo response of hematopoietic niche to clinically cues*, *whereas conventional cell cultures do not*
Thon et al., Blood 2014 [56]	*BMC biology*	PDMS/Soft-lithography	Syringe pump	a- and h-MK/Alginate; Matrigel	Static 3D culture/Methods: Cell morphology; Cell differentiation	*Co-culture-perfused 3D hydrogel matrix*
	*Development of a scalable*, *human-induced pluripotent stem cell-derived MK-compatible PLT microbioreactor*					*Provides a physiological BM environment in vitro as in vivo*, *improving platelet production compared to conventional cultures*
				h-UVEC/Fibronectin-coated surface		
Bruce et al., PLoS One 2015 [57]	*Modeling pathological BM*	PDMS/Soft-lithography	Syringe pump	h-abnormal lymphoblastic cell (ALL); h-MSC; h-OSB/Type I collagen	Static 2D culture	*Co-culture in perfused 3D hydrogel matrix*
	*Drug assay*				Static 3D culture/Methods: Chemotherapeutic assay; Cell-tissue interaction; Cell viability	*Provides characteristics in vitro based on BM physiology and structure in vivo*, *improving the investigation of the effects of cell-cell and cell-matrix interactions on the initiation and progression of cancer compared to static 2D and 3D culture*
	*Development of a 3-D microfluidic triculture model in a biomimetic manner*					
Zhang et al., PLoS One 2015 [58]	*Modeling pathological BM/BM niche mimicking*	PDMS/Soft-lithography	Syringe pump	h-OSB; h-plasma cell myeloma (MM)/OSB-generated	Static 3D culture/Methods: Cell viability; Cell proliferation; Biochemical analysis	*Co-culture in perfused 3D OSB-generated matrix*
						*The 3D microfluidic provides a microenvironment based on the perfused culture of MM in ECM OSB-generated capable of improving the mimic of MM-endosteal niche interactions*, *replicating the multicellular MM tumor niche with perfusion as an important microenvironmental factor and improving the maintenance of long-term co-culture*
	*Development of a 3D device for MM-bone marrow stromal cells long-term co-culture*					
Wuchter et al., Cell Tissue Res 2016 [59]	*BMC biology*	PC; PMMA; COC/Injection molding; hot embossing	Cassette pump	h-HPC; h-MSC/Type I collagen-coated surface	Dynamic 2D culture/Methods: Phenotypes analysis; Cell differentiation;	*Co-culture in perfused 3D MSC-generated matrix*
	*Development of a microfluidic 3D model system of the human hematopoietic stem cell niche*					*Provides an environment able to creating a more physiological environment for HSPC when compared to 2D co-culture*, *preserving its stem cell properties and improving the maintenance of stem cell culture for longer*
Torisawa et al., Tissue Eng–Part C Methods 2016 [60]	*BMC biology*	PDMS/Soft-lithography	Syringe pump	a-BMC/Type I collagen	Static 2D culture/Methods: Phenotype analysis; Cell viability; Radiation exposure;	*Co-culture in perfused 3D hydrogel/bone powder matrix*
	*Drug assay*			Demineralized bone powder		
	*Cultivating living marrow with a functional hematopoietic niche in vitro*					*Provides a bone marrow-based 3D model able to mimic the hematopoietic niche and the cell behavior as in vivo*, *including the improvement of the hematopoietic process such as hematopoiesis and the responses to drugs and radiation compared to conventional cultures*
Miller et al., Biotechnol Bioeng 2016 [61]	*BMC biology*	Silicone; PMMA; PC/Laser cutting; Milling	Gravity-induced flow	h-abnormal megakaryoblastic cell (CML)/PGMatrix hydrogel	Static 2D culture/Methods: Cell viability;	*Perfused 3D hydrogel matrix*
	*Describing the design and operation of a 14 chamber multi-organ system*					*Provides a microphysiological system capable of mimicking cellular behavior as in vivo*, *improving the maintenance of viability in vitro compared to conventional cultures*
Zheng et al., Adv Heal Mater 2016 [62]	*Modeling pathological BM*	PDMS/Soft-lithography	Reservoir	h-UVEC; h-abnormal promyeloblastic cell (APL); abnormal bone marrow cells (CML, HL)/h-Stromal marrow cell/Type I collagen	No control culture/Methods: Cell motility; Angiogenesis analysis	*Co-culture in 3D hydrogel matrix*
	*Using a 3D biomimetic model to study leukemic cell-induced bone marrow angiogenesis*					*Provides a microengineered 3D microenvironment able to promote the angiogenesis induced by different types of leukemic cells*
Houshmand et al., Tissue Eng–Part C 2017 [63]	*Modeling pathological BM*	PDMS/Soft-lithography	Peristaltic pump	h-abnormal erythroblast cell (ERY); h-MSC/Demineralized bone matrix	Static 2D culture/Methods: Phenotype analysis; Cell proliferation; Cell morphology; Chemotherapeutic assay; Cytotoxicity assay	*Co-culture in perfused 3D scaffold*
	*Drug assay*					*Provides a 3D mimicked AML microenvironment capable of studying the effect of stromal cells and bone marrow niche on the viability of leukemic cells*, *improving cellular proliferation when compared to conventional cultures and predicting their response to drugs as in vivo*
	*Designing of a perfusioned 3-D microenvironment to mimic the acute myeloid leukemia for molecular study and drug screening*					
				Type I collagen		
Sieber et al., J Tissue Eng Regen Med 2018 [64]	*BMC biology*	PDMS/Soft-lithography	Peristaltic pump	h-MSC; h-HSPC/3D hydroxyapatite ceramic	Static 2D culture/Methods: Cell differentiation; Phenotype analysis; Gene expression analysis	*Co-culture in perfused 3D scaffold*
	*Generation of a versatile*, *bone in vitro culture system mimicking the human bone marrow and niche biology*					*The BM-on-a-chip that integrates a 3D structure*, *and a physiological flow provides a model of BM niche environment capable of long-term culture of HSPC*, *improving the cell behavior in vivo when compared to conventional methods*
Kotha et al., Stem Cell Res Ther 2018 [65]	*BMC biology/BM niche mimicking*	Type I collagen/Injection molding	Gravity-induced flow	h-Stromal marrow cell; h-MSC /Type I collagen	No control culture/Methods: Cell migration; Cell adhesion; Phenotypes analysis; Cell-cell interaction; Gene expression	*Co-culture in perfused 3D hydrogel matrix*
	*Development of an engineered human vascular marrow niche*					*Provides a microenvironment able to study the mechanisms behind dynamic spatial and temporal cell-cell interactions within the vascular niche in both healthy and disease-remodeled marrow spaces*, *improving the understating about hematopoietic cell adhesion*, *transmigration*, *and engraftment*
				h-UVEC; h-monocytes; h-HSPC; abnormal bone marrow cells (AML)		
Marturano-Kruik et al., Proc Natl Acad Sci U S A 2018 [66]	*BMC biology/BM niche mimicking*	PDMS/Soft-lithography	Syringe pump	h-MSC; h-UVEC/Decellularized bone scaffold	Static 2D culture	*Co-culture in perfused 3D scaffold*
	*Mimicking the human bone perivascular niche*				Dynamic 3D culture/Methods: Vasculogenesis assay; Histological analysis; Gene expression; Chemotherapeutic assay	*Provides a 3D environment capable of mimicking the native structure and surface properties of bone marrow*, *interstitial flow*, *controlling the transport of nutrients*, *metabolites*, *and oxygen*, *improving the angiogenic processes in an in vitro model*
Aleman et al., Small 2019 [67]	*Modeling pathological BM*	PDMS/Soft-lithography	Peristaltic pump	h-BMNC/HA gelatin	No control culture/Methods: Cell viability; Phenotype analysis; Cell quantification	*Co-culture in perfused 3D hydrogel matrix*
	*BMC biology*			h-HSPC; h- abnormal bone marrow cells (HL, AML);		*Provides a biomimetic physiologic environment*, *improving the study of the interactions of distinctive HSPC with different niches*, *under conditions of both health and disease*
	*Development of a human BM niche-on-a-chip (NOC) platform with an integrated recirculating perfusion system*					
McAleer et al., Sci Transl Med 2019 [68]	*Modeling pathological BM*	PMMA; PDMS/Laser-cut	Gravity-induced flow	h-abnormal myeloblastic cell (AML); h-abnormal megakaryoblastic cell (CML)/Alginate	No control culture/Methods: Cell viability; Cell density; Enzymatic assay; Chemotherapeutic assay; Cell proliferation; Cell quantification	*Co-culture in perfused 3D hydrogel matrix*
	*BMC biology*					*Provides a mimetic system able to investigate anticancer drug efficacy and off-target effects*, *improving the response of the cell and effectively demonstrating the effects on cancer cells observed clinically*
	*Drug assay*					
	*Mimicking the multi-organ system*					
Herland et al., Nat Biomed Eng 2020 [69]	*BMC biology*	PDMS/Soft-lithography	Peristaltic pump	h-UVEC/Type I collagen- and fibronectin-coated surface	No control culture/Methods: Phenotype analysis; Cell quantification	*Co-culture in perfused 3D hydrogel matrix*
	*Drug assay*					*Provides a mimetic human system in vitro suitable for drug discovery*, *regulatory assessment*, *toxicological evaluation*, *and personalized medicine*, *improving the therapeutic development by enabling the more effective design of drug regimens for future phase-I clinical trials*
	*Designing of a first-pass model of human drug absorption*, *metabolism and excretion*					
				h-HSPC/Type I collagen fibronectin		
Chou et al., Nat Biomed Eng 2020 [70]	*BMC biology*	PDMS/Soft-lithography	Peristaltic pump	h-HSPC; h-HSPC abnormal (SDS); h-MSC/Type I collagen	Static 2D culture	*Co-culture in perfused 3D hydrogel matrix*
	*Modeling pathological BM*				Static 3D culture /Methods: Cell proliferation; Cell differentiation; Cytotoxicity assay; Chemotherapeutic assay; Radiation exposure; Phenotype analysis	*Provides an in vitro biomimetic model of vascularized human BM that improve the maintenance*, *proliferation*, *and cellular functions*, *enabling the recapitulation of many clinically relevant features of BM pathophysiology in response to clinically relevant exposures to drugs and radiation*
	*Drug assay*					
	*Development of a vascularized human BM-on-a-chip*			Fibrinogen		
				h-UVEC/Type I collagen- and fibronectin-coated surface		

^a^*BMC biology*; General descriptor for clustering publications that aim to mimic the BM environment without distinction of niches and/or represent the processes and phenomena associated with BM cells such as proliferation, differentiation, migration, hematopoiesis, et cetera

^b^*Modeling pathological BM*; General descriptor for clustering publications that aim to mimic the abnormal cell behavior in BM microenvironment *in vitro*

^c^*Drug assay*; General descriptor for clustering publications that report the cell's response to drugs, radiation, chemotherapeutics, et cetera

^d^*BM niche mimicking*; General descriptor for clustering publications that aim to mimic specific niches *in vitro* considering their physiology and architecture

^e^The material has not been identified

BMC, bone marrow cells; prefix h-, human cells; prefix a-, animal cells; MM, multiple myeloma; AML, acute myeloid leukemia; ALL, acute lymphoblastic leukemia; CML, chronic myelogenous leukemia; APL, acute promyelocytic leukemia; HL, histiocyte lymphoma; ERY, erythroleukemia; SDS, Schwachman–Diamond syndrome; UVEC, umbilical vein endothelial cells; MSC, mesenchymal stem cells; OSB, osteoblasts; MK, megakaryocytes; HPC, hematopoietic progenitor cells; HSPC, hematopoietic stem progenitor cells; BMNC, bone marrow mononuclear cells; HA, hyaluronic acid

### OoC applications

One of the objectives of this review was to identify the main applications of the BM mimicking devices reported in the literature. Four broad categories of applications have been identified: a) BMC biology [51, 55, 56, 59–61, 64–70], defined as a descriptor for classifying publications that aim to mimic the healthy BM environment without distinction of niches and/or represent the processes and phenomena associated with BM cells such as proliferation, differentiation, migration, and hematopoiesis. In this group, the main cells regulating BM functions were considered, including mesenchymal stem cells, hematopoietic stem progenitor cells and osteoblasts; b) Modeling pathological BM [50, 52–54, 57, 58, 62, 63, 67, 68, 70], defined as a descriptor to classify publications that aim to mimic the pathological microenvironment and the behavior of abnormal cells in a BM-on-a-chip; c) Drug testing assay [50, 52, 54, 55, 57, 60, 63, 68–70], defined as a descriptor to classify publications that report the cell's response to drugs, radiation, chemotherapy and other stimuli; and d) BM niche mimicking [51, 53, 58, 65, 66], defined as a descriptor to classify publications that aim to mimic specific niches *in vitro* taking into account their physiology and architecture.

As the variety of applications suggests, these devices have a broad of spectrum of uses. Though studying healthy BM cells and microenvironment is the main focus of most of the articles selected for the systematic review, the large number of publications addressing the abnormal BM microenvironment indicates that the study of BM-affecting pathologies is another emerging biomedical application for OoC devices. We have identified the publications that assessed multiple myeloma (MM) [53, 54, 58], acute lymphoblastic leukemia (ALL) [57], acute myeloid leukemia (AML) [50, 52, 67, 68], chronic myeloid leukemia (CML) [61, 62, 68], and other cells. The common goal of these publications was contributing to the understanding of the physiopathology of BM diseases and investigation of new treatment protocols for people with these diseases.

From the physiological point of view, one of the important aspects of BM structure are the niches: the regions with distinctive properties and cell types [6] working in an integrated manner to ensure the function of niche cells. Regarding the category ‘BM niche mimicking’, it should be noted that only five of the selected publications considered the BM niches and none of the proposed devices addressed all the three niches. The great majority of the devices represented specific regions of BM, e.g., close to the endosteum (endosteal niche) [53, 58] or around the blood vessels (perivascular niche) [51, 65, 66]. The development of devices that mimic only one region of the BM is most likely related to the difficulties in representing the distinct physical, chemical, and biological characteristics of BM [71]. Nevertheless, mimicking individual niches can help to understand the processes governed by the specific characteristics of the given regions and explain some aspects of the cellular behavior in the BM environment. From this perspective, it should be noted that two papers included in the review assess the influence of the endosteal niche on the response of abnormal cells [53, 58] and one assesses the behavior of BM cells in a 3D environment with an oxygen gradient, and mimics the perivascular niche [66].

On the other hand, mimicking only a given region certainly affects the obtained information since in this case, the devices cannot represent various interactions that exist between BM niches.

It is interesting to note that the developed devices mimicking BM in some cases, are part of a more complex system. Five of the selected publications [50, 52, 61, 68, 69] concern the devices that in one way or another attempt mimicking BM as a part of a microphysiological model of the human body; the devices built for this purpose are known as the body-on-a-chip or multi-organ system devices.

### Material and fabrication techniques

The development of OoC devices involves several challenges associated with their fabrication, including the costs and time required, the geometry and size of the channels, and surface properties. The selection of suitable materials and fabrication techniques is one of the most important steps in the development of OoC devices.

#### Fabrication materials

The selection of the most appropriate materials for each application of OoC depends on some factors that shall be taken into account in BM mimicking devices.

The first one concerns the biocompatibility of materials, needed to guarantee an adequate cellular response in a specific application [72].

The second one is the maintenance of the long-term culture, which is vital in the studies of hematopoietic processes [73] and biological processes associated with mesenchymal cells (*e*. *g*. differentiation and ECM secretion).

Next factor is gas exchange governed by permeability. Standard cell cultures require stable and well-defined O_2_ and CO_2_ concentrations; any variations in these parameters can be harmful to the cells.

One more factor concerns chemical products that may form at the interface pharmaceutical product/material. These chemical products can be converted into signals that influence the cellular response to a given clinical treatment [74]. This is important, for instance, when BM chips are used in drug testing.

And one more factor is related to the optical properties of the material. The imaging techniques that are usually used for data collection (*e*.*g*., fluorescence microscopy, confocal microscopy, phase-contrast microscopy) require the use of transparent OoC devices that can be monitored without disassembling.

The present review showed that PDMS, PMMA, silicon, PC, and silicone were the main materials used to create BM channels in OoC devices. Polymeric materials were used more often, suggesting that they have the properties required for in BM mimicking OoC devices [75]. Compared to brittle glass and silicon materials that require non-trivial sealing protocols, thus making the fabrication process expensive and complex, polymers offer cost-effective manufacturing and allow creating high- quality low-cost products.

The review has shown that PDMS was the most commonly used polymer material for the construction of BM channels in OoC devices. PDMS is a biocompatible material that can be sterilized in an autoclave and is easy to use in microengineering, explaining its widespread use in the production of OoC devices [76]. Furthermore, PDMS has remarkable elasticity, low production costs [77, 78], and high oxygen permeability, which allows using it for long-term cultures. The translucency of the material facilitates the use of imaging technologies, e.g., confocal and fluorescence microscopy for the biological characterization of the environment [79].

In the articles that were analyzed in this review, PDMS was used in several applications for *in vitro* mimicking of BM, including a) assessment of normal and abnormal behavior of BM cells in drug presence [50, 52, 54, 55, 57, 60, 63, 68–70], and b) creation of long-term cultures (more than 20 days) [53, 58, 64, 69, 70].

At the same time, even though it is the first-choice material in the analyzed articles, some characteristics of the PDMS are disadvantageous for its use in BM mimicking OoC devices. For example a) the absorption of the medium culture and drug components; b) formation of bubbles; and c) distortion of the device’s geometry [77, 80].

Recently, efforts have been made to identify and test new materials able to replace or modify the PDMS for use in BM OoC devices [81–85]. To overcome the disadvantages mentioned above some authors proposed the use of coating methods and design adequation [84, 85].

Among the alternative materials identified in this systematic review, PMMA appears to be a good option and is cited in three publications. PMMA is the acrylate-family polymer known for fracture resistance, translucency, and elasticity [86], as well as high chemical resistance [87]. In addition, PMMA is a low-cost and easily manufactured material [88]; it is also less hydrophobic than PDMS or other polymers used in microfluidic devices, thus ensuring a lower absorption of hydrophobic molecules crucial characteristics for the preservation of cell culture.

#### Fabrication techniques

The choice of fabrication methods is based on material properties. For the devices made of PDMS as the main material, the conventional soft-lithography was used [51–53, 55–58, 60, 62–64, 66, 67, 69, 70]. Soft-lithography is a technique based on conventional photolithography for fabrication or replication of structures made of elastomers. It is generally used to produce micro- and nanostructures [78, 89] and its low cost and versatility makes it the most common technique in the fabrication of microfluidics devices, since it allows building different planar and non-planar surfaces and 2D and 3D structures with a resolution of up to 30 nm and a minimum size of 10 to 100 nm [90, 91].

The main drawback of this technique is that it is time-consuming and requires highly trained and specialized personnel for the operation of the equipment [52].

Besides soft-lithography, two other fabrication techniques were used a) laser cutting [61, 68]; and b) injection molding [54, 59, 65]. Process automation makes the laser cutting and injection molding relatively simpler fabrication methods for the operators compared to soft-lithography. However, fabrication of the master mold is very expensive because of the high pressures and temperatures involved. The most commonly used polymers are the most rigid ones, such as PC and PMMA.

### Flow mechanisms for device perfusion

One of the most important characteristics of OoC systems is that they allow mimicking the cell microenvironment while taking into account the fluid flow in the same way as it is present in living organs, thus expanding the scope of studies and helping to understand the phenomena within this microenvironment. Using a fluid flow allows modelling physiopathological conditions in human organs on a microfluidic device [25, 26]. The stimulation caused by a fluid flow can induce biological cell responses through a mechanism known as mechanotransduction [92]. The mechanical shear stresses generated by the fluid flow also influence several cellular processes, e.g., proliferation and differentiation of human osteoblasts [93], adhesion of leukemic and hematopoietic progenitor cells [94], osteogenic differentiation of MSCs [95, 96], emergence of HSCs via endothelial-to-hematopoietic transition [97], and endothelial cell behavior [98, 99].

Finally, it is important to mention that almost all studies included in the review take the fluid flow into account. Fluid flow and recirculation are essential for a realistic representation of the behavior of cells. In the BM microenvironment, interstitial fluid constantly stimulates the cells and induces cellular proliferation and differentiation [96, 98]; it also supplies the cells with oxygen, nutrients, and growth factors and removes the metabolic products from the cells.

Considering the importance of fluid flow in BM cell processes, one of the guideline questions outlined in this review was: Which mechanisms are used for device perfusion? The analysis of the selected papers identified two main mechanisms: a) active flow; and b) passive flow.

#### Active flow mechanisms

The syringe and peristaltic pumps were the most used active flow mechanisms in the revised publications. These mechanisms comprise systems containing a pump, a culture medium reservoir, and the connecting pipes between the device and reservoir. There were identified some differences between these systems, e.g., syringe pumps produce unidirectional flow, not allowing fluid recirculation, while peristaltic pumps allow recirculation. Because of the recirculation of the culture medium, peristaltic pumps can better mimic the physiology of the cellular microenvironment and, hence, are more suitable for the studies of the pharmacokinetics and pharmacodynamics of a drug in the human body [50, 100].

Though the pumps are the most used approach for fluid flow induction, they have some disadvantages. One problem related to the use of peristaltic or syringe pumps or tube-connected devices is that these systems can generate the so-called “air bubbles”, which are harmful to cells and must be avoided to maintain the integrity of experiment [52, 101]. As reported in some studies included in this review [50, 66], to overcome this drawback and prevent the bubbles, a “bubble trap” is usually attached to the system. The “bubble traps” require the use of a vacuum system, hydrophobic-coated surfaces, membranes, and pressurized fluids [102]. The “trap” is based on the floating of air bubbles associated with their low density. The bubbles are collected in a compartment that separates them from the fluid, allowing regular flow and preventing the cells from damage [101].

#### Passive flow mechanism

Taking the difficulties presented by active flow mechanisms into account, some studies included in the review used passive flow mechanisms, such as gravity-driven flow [52, 61, 65, 68]. This mechanism involves the use of fluid reservoirs located at different heights. In this case, gravity generates a flow proportional to the elevation and the density of the fluid [100]. This low-cost method prevents the formation of bubbles, is simple in operation, and generates flows with physiological shear stress on the cells [61]. Though having many advantages, the renewal of the culture medium in gravity-generated flow systems can be a problem. This can lead to recirculation of the culture medium with fewer substances and more waste from cell activity, which may lead to cell death and prevents long-term experiments [50].

It is noteworthy that through the development of OoC devices capable of growing cell culture under perfusion of culture medium, several studies were able to generate the dynamic BM microenvironment *in vitro*. Several *in vivo* characteristics of the BM were mimicked using different flow generation techniques: cellular stimuli created through physiological shear stress generated by the fluid [56, 58]; regulation of BM physiological parameters through fluid perfusion [55]; *in vitro* generation of flow within the medulla with physiological interstitial velocities [57, 66]; and creation of a physiological vascular system, including the supply of oxygen and nutrients and the removal of metabolic products [70]. This indicates that perfused cell culture *in vitro* can generate more realistic environments for cell culture, thus improving understanding of specific phenomena that occur in the BM.

### Materials and cells for mimicking the BM microenvironment

BM is one of the most complex and largest organs in the human body and the main hematopoietic organ, responsible for the production of different cell types, e g., erythrocytes, granulocytes, monocytes, lymphocytes, and platelets [103]. The BM ECM is a non-cellular structure composed of many proteins, such as collagen, laminin, fibronectin, and fibrinogen, which provides essential physical support for tissue integrity, elasticity, and the hematopoiesis process. In addition, BM ECM provides a biochemical environment for cellular growth, differentiation, and maintenance [3, 104, 105]. It is also reported in the literature that cytokines, chemokines, and ECM proteins provide stimuli and interactions for cell homing through specific biochemical signals and regulate some HSCs functions [104, 106].

The main function of BM microenvironment is to maintain homeostasis by providing signals that regulate and support the differentiation of HSCs in the progenitor blood cells and the subsequent proliferation of these differentiated cells, leading to the production of billions of blood cells [107].

A key aspect of understanding how to develop more realistic BM-mimicking devices is the evaluation of material properties and cell types of this microenvironment. Improved BM microenvironment representation is a central issue for *in vivo* mimicry. One of the most important aspects mentioned in the selected publications was how BM substrate/ECM is represented. Besides that, the cells selected for microenvironment composition and the culture are also extremely important in mimicking the BM. Based on that, the studies mimicking BM can be divided into two categories that include the ECM representation and cell composition representation, respectively.

#### BM microenvironment

Based on the different studies included in this review, three models representing the BM were identified: hydrogel/gel; scaffolds; and ECM-secreted (Table 2).

**Table 2:** Materials used for mimicking the bone marrow.

Material types and structure		Gel model	Scaffold model	ECM secreted
3D matrix	Surface coating			
Type I collagen [54, 57, 62, 65]	Type I collagen [59]	**•**		
Alginate [50, 52, 56, 68]		**•**		
Matrigel [56]		**•**		
Type I collagen/fibrinogen [70]		**•**		
Type I collagen/fibronectin [69]	Type I collagen/fibronectin [69, 70]	**•**		
PGMatrix [61]		**•**		
Fibrin [51]		**•**		
HA Gelatin [67]		**•**		
Bone powder/type I collagen [55, 60]		**•**		
Bone matrix/type I collagen [63]			**•**	
Hydroxyapatite [64]			**•**	
Decellularized bone [66]			**•**	
Polystyrene [66]			**•**	
Ossified tissue [53, 58]				**•**
	Fibronectin [56]	**•**		

The first approach used to mimic the BM microenvironment was the hydrogel model. A hydrogel can be defined as a solid lacking a macroporous design [70]. The hydrogel is a polymerizable material that permits forming transparent 3D structures. In the ECM formation, the polymerization process of the 3D network influences the interactions between the cells and the matrix in this microenvironment [108]. That is, the cells interact with the cellular microenvironment similarly to their *in vivo* counterparts. This interaction influences the cell's phenotype and the factors they secrete in ECM [109]. In addition, the transparency of the material permits obtaining high-resolution images of the culture [62].

Chemical and mechanical properties of hydrogels depend on the type of hydrogel used in cell cultures [110]. Type I collagen, alginate, Matrigel [56], hyaluronic acid [67], fibronectin [69], and fibrin [51] are some of the protein types and ECM components that compose natural hydrogels [8]. The use of natural hydrogels provides a suitable microenvironment for the cultivation of BM cells, keeping their functions similar to those *in vivo* [57]. However, different production protocols of these components lead to variation in the compositions, making it difficult to predict and standardize the effects of the hydrogel matrix on cell behavior [110]. In the gel/hydrogel models, the most used components mimicking the BM environment were a) type I collagen-based hydrogel [54, 55, 57, 60, 62, 65, 69, 70] and b) alginate-based hydrogel [50, 52, 56, 68]

Type I collagen is fibrillar collagen. Probably it is the most studied and abundant collagen type. Its Young’s modulus depends on a density. Different studies have reported the production of 3D collagen structures with Young’s modulus values around 10 Pa (0.5 mg/ml) [111], 300 Pa (2 mg/ml) [111, 112], and 1200 Pa (4 mg/ml) [111]. Type I collagen with different densities can be used to mimic BM niches with different stiffness because its mechanical properties and their control methods are well known. Nevertheless, the low mechanical strength of collagen gels results in their significant contraction caused by the cells, and this is an important drawback of collagen use in systems without geometric constraints [113].

On the other hand, alginate hydrogel Young’s modulus ranges from 100 Pa (relatively elastic) to 10 kPa (moderately stiff) [114, 115] making it more suitable for mimicking the BM because BM Young’s modulus ranges from 0.1 to 1 kPa [1]. For example, in cultures of non-adherent abnormal cells, such as myeloblastic lineage cells, alginate provides a more realistic environment than Matrigel, retaining leukemic cells and improving their response [50]. In addition, when used as an ECM, alginate hydrogel provides greater cell growth compared to standard cell culture protocols [1].

Another way of representing ECM is by structures referred to as scaffolds; the scaffolds can be made of hydroxyapatite, polystyrene, and bony structure (Table 2). Scaffolds are well-defined macroporous 3D structures with properties similar to those found *in vivo* [116]. Scaffolds are characterized by their geometry, mechanical properties, and composition. The pores, the size, architecture, fabrication methods, and used material govern the properties of the scaffold [117]. In the reviewed publications, the scaffolds were made of synthetic materials [64, 66] or bony structures [63, 66].

Scaffolds can mimic trabecular bone, especially its structural and mechanical properties, allowing, therefore, to represent a spongy bone and BM niches [64]. This permits modelling the endosteal niche located near the trabecular bone that regulates the behavior of human hematopoietic cells [6].

Unlike the hydrogel-formed structure, the use of the scaffolds has some technical drawbacks. The materials commonly used to create scaffolds, such as hydroxyapatite, polypropylene, and polystyrene, may not be transparent [63, 64, 66]. Since a large part of the analysis techniques is based on light microscopy, opacity makes it difficult or impossible to view and record images, affecting the assessment of the device's effectiveness.

Finally, in addition to studies pointing out the hydrogel/gel and scaffolds, some studies have used living cells to produce the ECM by cultivating the cells on the surface of the device itself, creating specific niches of BM microenvironment [53, 58]. Using niche cells to generate the microenvironment through the secretion of ECM, growth factors and cytokines produce a matrix with chemical and mechanical properties that maintains the cellular behavior similar to *in vivo*.

For cell cultures on the device surfaces [56, 59, 69, 70], specific coatings were used to mimic the surface properties of the cellular environment of organs and tissues. The coating improves the cell adhesion and generates a phenotypic representation as expected *in vivo*, ensuring a realistic cell behavior in a given environment [118].

#### BM cell types and cellular culture

A wide variety of cell types was used to constitute the bone marrow microenvironment. Mesenchymal stem cells, osteoblasts, and endothelial cells are some of the reported cell types, in addition to hematopoietic [59, 65, 67, 69, 70] and leukemic cells [50, 52, 57, 61, 62, 65, 67, 68]. Other studied cell types are given in Table 1.

The studies have evaluated both co-cultures and single-cell cultures. As BM is a very complex biological organ, it is essential to understand the functioning of its regions to choose the best cell culture approach [6]. The culture of one cell type provides a more manageable environment for *in vitro* testing, making it possible to analyze the collective behavior of specific cell types under well-defined physiological conditions. However, the *in vivo* cell environment is composed of several cell types and the cell-cell and cell-ECM interaction are determinant for cellular behavior. For example, cells that are present in the endosteal, central, and perivascular niches play key roles in stem cell behavior, regulating differentiation into red and white blood cells that will migrate into the bloodstream [119, 120]. A simulation of this complex environment *in vitro* is challenging for researchers, as regions with different mechanical and biological properties need to be reproduced in the same device [3], requiring the adjustment of the culture medium, oxygen supply, fluid flow, and mechanical properties in a complex microfluidic system. Nevertheless, most of the publications in the review use the co-culture approach to represent the cellular microenvironment of BM. This suggests the evolution of technologies and methods of cell culture that allow implementing this model with the *in vitro* systems. However, some screened publications used single-cell cultures to represent in a simplified way a phenomenon that in reality occurs in a complex BM environment.

Though BM environment is a dynamic system governed by cell-cell and cell-ECM interactions, integrating the ECM and cells properties in the OoC devices analyzed in this systematic review allowed detecting various cellular behaviors and phenomena associated with BM: the regulation of HSP and HPC cells in the hematopoietic microenvironment by interaction with niche cells, such as osteoblasts, perivascular endothelial, and perivascular stromal cells [55]; the preservation of MM cells in a 3D ossified tissue constructed by a human osteoblast cell line (endosteal niche) [53, 58]; the effect of chemotherapeutic drugs on abnormal cells in a 3D environment with co-cultivation of BM cells [57, 63]; the hematopoiesis process in a 3D BM model based on collagen hydrogel and bone powder [60]; the long-term culture of HSPC in co-culture with MSC in a 3D scaffold that mimics trabecular bone [64]; the regulation of normal and abnormal HSPC behavior in a 3D multi-niche system [67]; and the mimicking of hematopoiesis processes in a 3D matrix in co-culture with MSC and HUVEC [70].

### Effectiveness of current OoC devices mimicking bone marrow

Over the years, the understanding of cellular processes and the clinical trial stages for drug development was achieved with the support of conventional cell culture platforms (2D and 3D static). Despite the relative success of this approach, it is important to stress that these platforms represented the cellular microenvironment in a simplified way [121]. It is also important to underline that the ideal *in vitro* cell culture representation must include two crucial aspects: the physiology and the structure of the cellular microenvironment. This can be achieved by ensuring three basic conditions in the devices: cell culture in a 3D environment, cell co-culture, and cell culture under perfusion.

Considering the above-mentioned basic conditions as advances in conventional cell culture methods, the articles included in this systematic review were classified into five groups according to the approach used: perfused 3D hydrogel matrix [50, 52, 61]; co-culture in 3D hydrogel matrix [51, 54, 62]; co-culture in perfused 3D hydrogel matrix [55–57, 60, 65, 67–70]; co-culture in perfused 3D scaffold [63, 64, 66]; and co-culture in perfused 3D cell-generated matrix [53, 58, 59]. To assess the effectiveness of these new design proposals, the studies integrated used control groups based on conventional platforms and a gold standard for evaluating the causal hypothesis: *static 2D culture* [50, 52, 57, 60, 61, 63, 64, 66, 70]; *dynamic 2D culture* [59]; *static 3D culture* [54, 56–58, 70]; and *dynamic 3D culture* [66]. Seven publications included in this review did not report a control group in their experimental analysis [51, 53, 55, 65, 67–69]. The detailed list of main evaluation technologies and cellular assays are given in S1 Table.

The evaluation of the device’s effectiveness was performed taking into account groups that were defined with the three basic conditions described above.

In order to better understand the influences of 3D matrix, co-culture, and the fluid flow on the reported results, an incremental evolution analysis was used: the articles were assessed in groups that represent the increase of the complexity of the device compared to the conventional method. These groups were classified as: a) static cultures of multiple cell types; b) perfused cultures of single cell types; and c) perfused cultures of multiple cell types.

#### Static cultures of multiple cell types

The first approach, static cultures of multiple cell types, was based in a 3D co-culture *in vitro* system without the presence of fluid flow. BM mimicking using this approach allowed reproducing the phenomena of vessel formation in the healthy BM [51] or the vessel formation induced by leukemic cell lines [62]. It is known that the presence of blood vessels in BM physiology is vital for the supply of nutrients, oxygen, and growth factors necessary for niche cell survival and regulation of hematopoietic cell behavior [5].

The region where these vessels are located in a healthy BM is called the perivascular region, or perivascular niche of the BM. With an objective to mimic this niche specifically, publications considered endothelial and stromal cells as basic components, since these are the cell types present in the native composition of the perivascular niche of the BM [8].

The simulated behavior of cell migration [51, 62] and adhesion [51], vasculogenesis [51], and angiogenesis [62] of niche cells were reported to be similar to *in vivo*; in addition, there was also the formation of vessels through the association between multicellular aggregates and fibroblasts sprouting in a primitive capillary plexus as it is detected in the microenvironment of BM.

Besides the study of cell behavior around the vascular region, the 3D co-culture approach also provides useful tools for understanding the response of tumor cells to the action of drugs. In a 3D matrix based on type I collagen, an experiment carried out to evaluate the chemotherapeutic effects on plasma cell myeloma reported a reduced action of drugs when the abnormal cells were cultured with bone stromal cells [54], resulting in increased viability (live/dead) of the cells. This phenomenon occurs because of a mechanism known as cell adhesion–mediated drug resistance caused by a) the direct MM–BM stroma cell adhesion; b) by MM-ECM adhesion; or c) by the secretion of a soluble factor [54]. Cell adhesion-mediated drug resistance is related to the increase of drug resistance mediators and avoiding the apoptosis process [122].

#### Perfused cultures of single cell types

Using the second approach, perfused cultures of single cell types, which is based on a perfused 3D system without co-culture, publications reported better results for toxicity response and cellular proliferation than those reported in conventional 2D control groups. Evaluating viability (toxicity and proliferation), myeloblastic cell lines showed greater sensitivity to drug exposure [50, 52] and megakaryoblastic cell lines were maintained in long-term culture [61].

The two previously mentioned approaches showed that a simplified BM mimicking with the *in vitro* system was able to represent the phenomenon that occurs *in vivo*. In other words, a system that uses the minimum characteristics essential to mimic a specific phenomenon can replicate it *in vitro*. The studies that use a 3D co-culture [51, 54, 62] or perfused 3D single-cell culture [50, 52, 61] consider the influences of two specific features of microenvironment on cell’s response, besides the ECM interactions, namely: a) cell-cell interactions; and b) fluid flow action.

Cell-cell and cell-ECM interactions play a key role in regulating cell functions and the microenvironment. These interactions occur through the so-called cell junctions and involve mediator proteins including cadherins and integrins that sense and respond to the external signals of the microenvironment [123].

The dynamic flow of the physiological fluid supplies nutrients, oxygen, and growth factors, and removes metabolic products from the cells. In addition, cellular behavior is influenced by flow-generated shear, which promotes a biological response of cells according to the mechanisms involved in cellular mechanotransduction [124, 125].

It is important to point out that the two approaches discussed so far have considered isolating the co-cultured cells in a static system or single-cell culture in a perfused system. Despite the important achievement in obtaining similar biological results as *in vivo*, the individual evaluation of these mechanisms is a simplified representation of the BM microenvironment and does not consider the dynamics of interactions and stimuli.

#### Perfused cultures of multiple cell types

**Fig 2:**
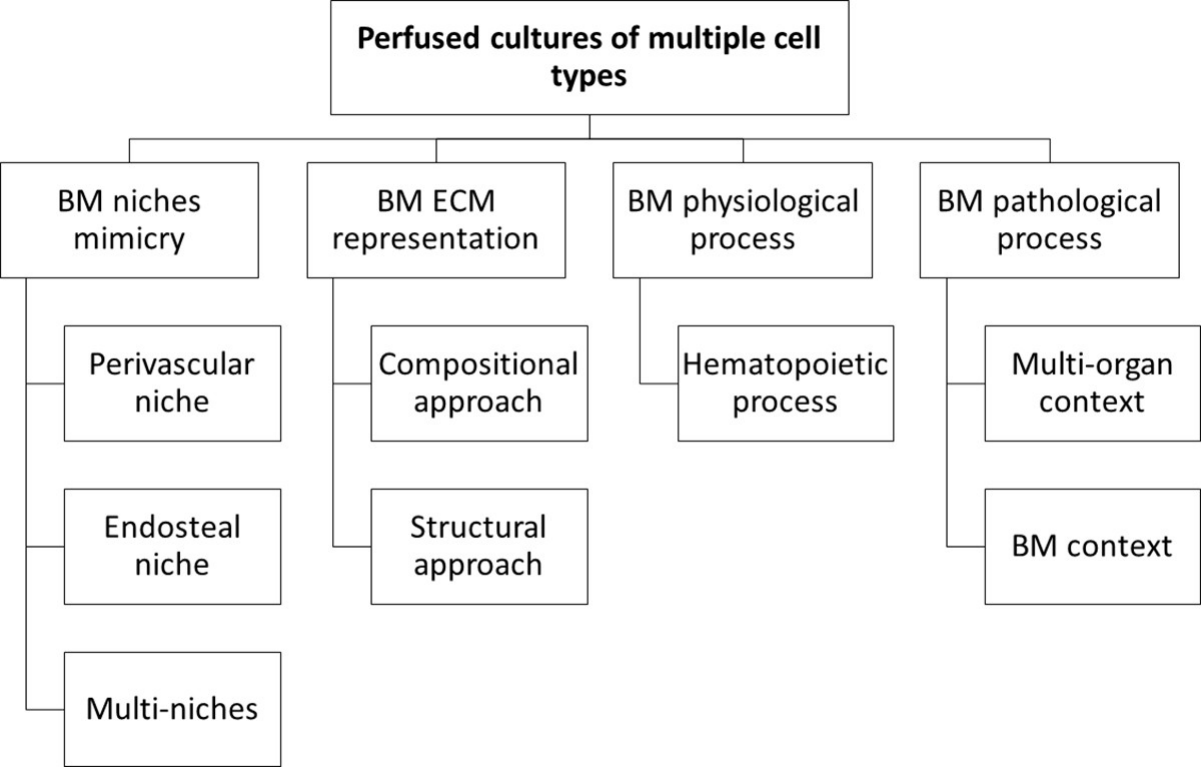
Organization and content diagram. Scope of Perfused cultures of multiple cell types section.

The third group of studies that attempted to overcome these issues and took into account all the three basics conditions for representation of BM environment *in vitro* was clustered: a 3D environment, formed by the proper matrix; a co-culture with specific bone marrow cell; and a perfused culture medium, as a source of oxygen, nutrients, and shear stress stimulation. In this systematic review, this group of studies was further subcategorized by specific applications and approaches: a) focusing in mimicry specific regions of BM; b) different approaches to BM ECM representation; c) studies of physiological process; and d) studies of pathological process. The organization and content of the subcategories in this subsection is presented below (Fig 2).

Considering a BM as an environment composed of specific and well-established regions, the use of perfused 3D co-culture approach allows a more integrated mimicking of bone marrow niches, specifically perivascular, endosteal, and their deconstructed combination.

Kotha et al. developed a 3D matrix based on hydrogel co-cultured with normal and abnormal cells that allowed the study of some important cellular processes around the perivascular niche: transmigration and the adhesion of hematopoietic cells in the healthy and pathological marrow [65]. Cell migration, cell-cell interaction, phenotypic analysis, and gene expression analysis were evaluated in this study. The results showed the device was able to represent the behavior of hematopoietic cells around the perivascular region and is a useful tool for the study of the dynamics of these cells in the niche.

Still focusing on the perivascular niche mimicking, Marturano et al. presented an OoC based on a 3D scaffold of decellularized bone co-cultured with endothelial cells and mesenchymal stem [66]. The generated physiological flow was regulated in order to generate an oxygen gradient along the scaffold. The authors evaluated the formation of vessels, gene expression and histology [66] and compared the results with two control groups: static 2D, to evaluate the influence of flow and its control over oxygen and nutrient supply; and dynamic 3D in a polystyrene scaffold, to evaluate the influence of surface properties. In both cases, the device’s performance was better than the control group. In the first case, this is associated with the two-dimensional arrangement and the non-physiological design of the supply of nutrients and oxygen in the conventional method. In the second case, the composition of the scaffold surface is made of a synthetic polymer, that does not provide the cells with the necessary structure for adhesion. In addition, the synthetic polymer modified the natural cell phenotype, and consequentially altered the physiological processes of the microenvironment [66].

Furthermore, the perfused 3D co-culture approach was able to mimic another vital niche in bone marrow: the endosteal niche. The endosteal niche is a region near the spongy bone composed basically of the bone itself and the stromal cells. This niche regulates several hematopoietic processes including the quiescence [126]. From the studies that aim to mimic the endosteal niche, it was observed that this approach also allowed the development of an OoC device capable of representing the interaction between abnormal cells and the niche microenvironment. In the work that used bone cells to generate an ossified 3D matrix, Zhang et al. studied how multiple myeloma cells behaved in this environment [53]. Based on the evaluation of cell proliferation and cell-tissue interactions, this study reported the formation of a 3D arrangement between abnormal cells and bone tissue. This arrangement is responsible for a phenomenon known as “cell adhesion-mediated drug resistance”, already studied in a work that used an approach without fluid flow for the representation of BM microenvironment [54].

In another study, using the same device to assess cell viability and proliferation compared to that reported by a 3D static culture, Zhang et al. demonstrated that perfusion in the endosteal niche cultivated with multiple myeloma cells is an important factor of the microenvironment that improves the maintenance of the long-term co-culture [58].

To conclude the discussion about the use of complete systems in niche mimicking, it shall be mentioned that Aleman et al. developed an innovative deconstructed model of BM niches. Using a perfused 3D gelatin-based matrix, the authors simulated the three niches of bone marrow in a single system, identifying them as perivascular (periarterial and perisinusoidal), mesenchymal, and osteoblastic. The niches were placed in separate chambers interconnected by channels and the study reported the interaction of normal and abnormal hematopoietic cells with the niche cells and the grow factors that they release; this device created a system for the study of the physiological and pathological BM [67].

It shall be noted that despite the importance of representing BM with niche formation, most publications that have developed 'perfused 3D co-culture' devices have modeled the microenvironment as 'a general bone marrow niche' [55–57, 60, 63, 64, 68–70].

In an *in vitro* BM system, there are three aspects that define whether the microenvironment model is suitable for the expression of the native cell phenotype: cellular culture as *in vivo*; cellular processes as *in vivo*; and cellular response as *in vivo*. The most basic goal of this system is to provide cells with a microenvironment that makes them behave as *in vivo*.

From this perspective, one way to evaluate the *in vitro* cell culture and to study how the environment stimulates the cells is through the modification of the arrangement of 3D BM models. As already discussed, the marrow microenvironment can be built considering specific biochemistry related to the matrix composition (*e*. *g*. proteins and biomolecules), or a specific structure related to the scaffold design (*e*. *g*. pore size and organization).

Concerning the compositional approach for the representation of ECM, Wuchter et al. developed an OoC device that generates a microenvironment that stimulates hematopoietic cells to express their phenotype as *in vivo* [59]. The cell co-culture was composed of the progenitor mesenchymal stem cells and the ECM was generated by the mesenchymal stem cells themselves, in a cellular network that provided the complete integration of progenitor stem cells [59].

Torisawa et al. created a biomimetic hematopoietic environment based on a 3D matrix composed of type I collagen and bone powder to study the cell maintenance and culture in a mimicked native environment. This 3D matrix was inserted (subcutaneous implantation) into an animal (rat), and after 8 weeks a complete BM niche (histological analysis) was generated [55]. All niche cell types were found in the BM 3D matrix, including hematopoietic cells, and cell behavior was maintained in long-term culture. The results demonstrated this system can generate a physiological response of the BM to radiation and anti-radiation drugs (cell viability) [55].

From the structural perspective, the maintenance of the behavior of cells and their long-term culture was achieved using a well-structured 3D scaffold formed by hydroxyapatite [64]. Hydroxyapatite mimics BM very well because it is analogous to the native bone structure. Using phenotypic and gene expression analysis in HSPC and MSC in co-culture, the authors reported that it was possible to replicate cellular behavior in an experiment performed on perfusion for 28 days. The comparison with a control group confirmed that modelling the BM ECM as a 2D monolayer is a poor and very simplified representation of a complex *in vivo* microenvironment.

Thus, the developed OoC-type devices mimic the BM microenvironment better and allow the maintenance of cell culture and replication of the *in vivo* cellular behavior. Furthermore, these systems also allow studying fundamental characteristics of cellular behavior: physiological processes.

Hematopoiesis is one of the most important physiological processes and is responsible for the development and maturation of blood cell elements including erythrocytes, leucocytes, and platelets.

Thon et al. developed a 3D system based on a hydrogel with cells stimulated by a flow-generated shear that improves platelet formation (cell differentiation analysis) compared to static 3D culture [56]. In this study, the vascular shear in microfluidic channels was the main contributor to the increase of platelet formation and for the reproduction of cell phenotype *in vitro* (cell morphology analysis)

In addition to the devices that evaluate the production process of specific cell lines in the BM, some devices seek to represent more broadly the entire hematopoietic process, mimicking what occurs in the medullary environment.

Using the previously developed device and experimental setup [55], Torisawa et al. evaluated the hematopoiesis processes in a BM-on-a-chip [60]. The results showed that after 13 days the blood cells production (histology analysis) and the hematopoietic cell behavior were maintained. This biomimetic environment simulated a physiological response of the BM to radiation and anti-radiation drugs [60].

The possibilities and results achieved with the BM OoC developed by Torisawa et al. characterize this device as an alternative to animal models [55, 60]. However, the results were based on animal cells; therefore, the translation into a human clinical trial would require the change of a protocol and a new approach to cell culture.

To advance in the study of BM for clinical applications, Chou et al., in an experiment cultivating one microfluidic channel with endothelial cells and the other with hematopoietic and mesenchymal cells in a hydrogel matrix formed by type I collagen and fibrinogen, developed an *in vitro* system with dynamic fluid flow reproducing hematopoietic processes [70]. This BM device improved the expansion and maintenance of differentiated hematopoietic cell functions when compared to static cultures. As reported by the authors [70], the improvement compared to conventional models has also been described for the toxicity response and drug treatment, thus, showing the advances that this device offers for the study of BM pathologies.

The study of pathologies in systems that mimic the three main aspects of a general BM niche can provide essential information for understanding how abnormal cells modify their surroundings and how it affects their response.

In human subjects, these studies can be made in two contexts: BM as a part of a complex system (multi-organ-on-a-chip); and BM as a complex system (bone-marrow-on-a-chip).

In the first context, McAleer et al. and Herland et al. developed a multi-system that improves the cells drug response [68] and provides a platform for drug screening [69]. Evaluating cell viability, cell density, chemotherapeutic assay, cell proliferation, cell quantification [68] and cell quantification, phenotypes analysis [69], both studies contributed to global understanding of the therapeutic effects of drug treatment in clinical trials.

In the second context, Bruce et al. mimicked a pathological BM environment with a tri-culture system (abnormal lymphoblastic cell, mesenchymal stem cell, and osteoblast) in a perfused type I collagen-based 3D matrix and recreated the intricate network of interactions (cell-cell and cell-ECM) that actually occur *in vivo* microenvironments. The reported conditions are correlated with the enhanced abnormal cell survival during the drug treatment [57] and underline that the leukemic cell lines show a higher resistance to chemotherapeutic drugs than cells in 2D models.

In other publications, the same phenomenon of drug resistance was reported, but with different approaches. Houshmand et al., using a macroporous model based on perfused demineralized bone 3D matrix with abnormal erythroblast cell and MSC co-cultured, found that leukemic 3D microenvironment protected the cell from drugs cytotoxicity [63]. Abnormal cells embedded in demineralized bone 3D matrix showed greater resistance to drug treatment than in 2D models.

From a clinical point of view, these results contribute to understanding why some therapies for treating hematological disorders have not yet achieved the expected efficiency. Drug screening on conventional 2D platforms predicts cell response in a non-physiological environment, disregarding the dynamic environment of interactions and stimuli that exist in the human body. Here, the effect of drugs is overestimated because the model does not consider a physicochemical barrier that protects the cells from drugs [53, 54, 57, 58].

An overall conclusion from the analysis of all articles included in this revision is that the devices developed so far for mimicking the BM are effective. All publications reported results that demonstrated the effectiveness of the devices, showing an improvement in relation to conventional models and shedding new light on the knowledge about cellular phenomena in BM. However, despite the improvements and considerable success in recreating *in vitro* BM environment, some limitations and challenges remain.

The main limitations identified in OoC devices in this systematic review were related to two vital features of BM that regulate and maintain the niche cells and hematopoietic cells: niches integration; and oxygen supply.

Regarding niches integration, it is already known that BM is formed by integrating three main regions vital for BM cell behavior: endosteal, central, and perivascular.

To develop an integrated system that incorporates the mentioned niches, cells-cells and ECM-cells interactions shall be considered, whether in the same niche or not. In this systematic review only one publication implemented the concept of several niches in the same OoC device [67]. However, these niches were not considered as an integrated system, since they were not physically interacting with each other and only the biochemical interaction was considered. Hence, even considering this interaction, which is also important for regulating BM cells, the lack of physical interactions and other cellular processes, e. g., cellular migration, poorly represent the dynamically complex BM microenvironment.

Here, two relevant papers shall be mentioned that are still under the peer review process and were not formally included in this revision. Nelson et al. developed a device where co-culture is done with main cells that compose the three niches: osteoblasts (specific endosteal cell), endothelial cells (specific perivascular cell), mesenchymal cells, and hematopoietic cells (niches cells) [127]. The culture was implemented in a hydrogel matrix based on collagen types I and IV, fibronectin, and lamina, all components existing in the BM microenvironment [8]. However, this study does not completely consider distinct niches of the BM, failing to represent the physiological and structural/spatial aspects.

Glaser et al. developed a system with distinct and interconnected chambers for the representation of the endosteal and perivascular niches. In the endosteal niche, culture was performed with osteoblasts and BM stromal cells. The perivascular niche was done with endothelial cells and BM stromal cells. Then, hematopoietic cells were grown in these niches [128]. The cells cultured in each mimicked niche were analogous to the main cell composition of the BM niches *in vivo* [129]. The cultures were implemented under perfusion, in a 3D matrix based on fibrin, with a hydrogel used to represent hematopoietic niches in the BM [8].

Despite the evolution in mimicking the BM niches shown in the studies by Nelson et al. and Glaser et al., they did not evaluate the hypoxia on the cellular response, failing to represent the physiological condition of oxygen concentration within the BM.

Oxygen conditions in BM have a key role in cells regulation, yet it was considered only in one study included in this review [66]. The BM is a system in a state of hypoxia or low oxygen concentration. It is also important to note that there is an oxygen gradient in the niches and that the oxygen concentration is higher in the perivascular niche and lower in the endosteal niche. It was reported [15] that this parameter regulates the behavior of hematopoietic cells, and its representation in a device mimicking the BM is essential to obtain a more realistic model. This issue was raised by Chou et al., who pointed out the possibility of changing oxygen concentration to study the behavior of HSC as a function of oxygen availability [70], and also by Houshmand et al., who reported that the absence of the hypoxia condition is a limitation in mimicking the BM niche [63].

### Limitations

Considering that a systematic review uses a process to identify comprehensively all studies for a specific focused question, our review presents limitations linked with the adopted approach.

Like many other systematic reviews, this review relies on a rather limited number of databases for the identification of potentially eligible studies. In this respect, it is worth to mention that the criteria for inclusion of studies into this review are also influenced by knowledge of the results of the set of potential studies. Other limitations are a) the use of studies published only in the English language; b) the time frame adopted (we considered published papers from January 1st, 2009 until July 24th, 2020); and c) absence of scores to assess the study quality.

As a concluding remark, we shall note that as any other research, systematic reviews have potential strengths and weaknesses. Nevertheless, we believe that the presented systematic review will help the readers to stay updated about recent developments to microfluidic devices developed for representation BM.

## Conclusions and perspectives

Understanding the limitations of conventional culture methods in mimicking the BM and motivated by the complexity of its microenvironment and its representations of niches, researchers developed *in vitro* culture devices of the OoC type with different levels of complexity, which repeat native behavior, processes, and responses of BM cells.

This integrative systematic review was guided by the following fundamental characteristics regarding the OoC device: a) device application; b) device design; c) fluid flow generation; d) ECM composition and cell types; and e) effectiveness of OoC. We have evaluated OoC technologies that simulate BM reported from 2009 to the present moment, focusing on device development and physical and biological features of the systems. Our research has shown that OoC technology has been widely applied to study BM in several applications: biological behavior of bone marrow cells; modeling of pathological bone marrow; bone marrow niche mimicking; and drug testing.

Regarding the material and fabrication process of the device, we found that the most common were the PDMS and soft-lithography, respectively. However, there are some limitations regarding the use of PDMS for building the device structure. The main limitations are the maintenance of the structure after manufacture and hydrophobicity, which is responsible for adsorption of components of culture medium and interactions with drugs. This subject requires further study for the development of new materials or treatment methods that meet the requirements of the experiments to reproduce the characteristics of BM during long periods in a reliable way.

The most used mechanisms for fluid flow generation were peristaltic and syringe pumps. These mechanisms provide a physiological flow as it occurs in BM, regulating the supply of oxygen, nutrients, and growth factors.

Collagen type I and alginate, which are natural polymeric materials, were the most used hydrogel types for reproducing the BM extracellular microenvironment. It is noteworthy that the 3D scaffold also was a solution for the representation of the BM, but in a macroporous approach. This approach is useful for representing the spongy bone and medullary cavities.

In order to evaluate the effectiveness of OoC, first, the devices were categorized by the advances compared to the conventional methods for cell culture. These groups were defined based on three main aspects of the *in vivo* BM: a) 3D arrangement of BM matrix; b) cellular co-culture; and c) presence of fluid flow. In the selected publications, we identified the devices that used co-cultured, perfused, and perfused in co-culture 3D BM matrix. The results showed that the three approaches improved the maintenance of the cellular culture, the long-term culture, the cellular behavior, the cellular process, and the cellular response to drugs compared to conventional 2D and 3D static culture.

However, despite these satisfactory results, the mimicking of BM has several structural and physiological limitations.

Because of the complexity of BM, composed of many different cell types and structures with different physical, chemical, and biological properties, virtually all approaches have focused on mimicking specific niches with specific cell types. None of the selected studies built the BM microenvironment *in vivo* integrating the three niches that form it. Besides that, the hypoxic condition and the distribution of oxygen are a scarcely explored aspect that was assessed in only one publication in this systematic review.

Using a simplified model to mimic BM may be sufficient to study specific and less complex problems, but because of the interaction of cells with the microenvironment and other BM cells failure to consider the three niches or the oxygen conditions leads to an incomplete mimicking of the organ *in vivo*.

Under these circumstances, the evolution and development of new devices mimicking BM in an OoC shall consider the following aspects: a) structural and spatial aspects (the composition of ECM and the distance between the niches); b) biological aspects (main cell types present in each niche); and c) physiological aspects (fluid flow providing oxygen, nutrients, and growth factors). Emerging fabrication techniques and alternative materials used to develop the devices contribute to the evolution of BM mimicry. One of the most important of them is 3D printing. This technique allows manufacturing highly complex devices and using several materials, including polymers, resins, and biomaterials. Regarding the materials, the focus is on the least hydrophobic ones that provide flexible manufacturing. PMMA is an example of such material.

The development of new OoC models should allow more complete studies of BM cells behavior and a more efficient system for studying the treatment of BM disorders.

## Supporting information

S1 Checklist PRISMA 2009 checklist. (DOC)

S1 Table Assessment technologies and assays. (DOCX)

S1 Appendix (DOCX)
