# Supplementary material for: Microfluidics and organ-on-a-chip technologies: A systematic review of the methods used to mimic bone marrow
Source: PLoS One. 2020 Dec 11;15(12):e0243840. doi: 10.1371/journal.pone.0243840 (PMC7732112; doi:10.1371/journal.pone.0243840)
Supplement: S1 Table — (DOCX) [file pone.0243840.s002.docx]

**S1 Table. Assessment technologies and assays.**

| Assays | Technologies |
| --- | --- |
| Cell viability | Fluorescence microscopy [1–7]  Flow cytometry [8,9]  Phase-contrast microscopy [10] |
| Cytotoxicity assay | Fluorescence microscopy [4,5]  Flow cytometry [11] |
| Cell morphology | Fluorescence microscopy [11,12] |
| Pharmacodynamic assay | Fluorescence microscopy [2] |
| Chemotherapeutic assay | Fluorescence microscopy [2]  Flow cytometry [8,11]  Luciferase assay [13]; Phase-contrast microscopy [10] |
| Phenotypes analysis | Fluorescence microscopy [1,7,11]  Confocal microscopy [14]  Flow cytometry [15,16]; PCR [17] |
| Cell proliferation | Fluorescence microscopy [11]  Flow cytometry [9,14,18][13,15,19][13,15,19] |
| Vasculogenesis analysis | Confocal microscopy [13] |
| Cell migration | Confocal microscopy [20]; SEM [19] |
| Angiogenesis analysis | Confocal microscopy [21] |
| Cell functions | Confocal microscopy [3] |
| Biochemical analysis | Confocal microscopy [3]  Flow cytometry [9] |
| Cell adhesion | Confocal microscopy [19]  Spectrophotometry [20] |
| Cell-cell interactions | Confocal microscopy [19] |
| Cell-tissue interactions | Confocal microscopy [18] |
| Cell differentiation | Flow cytometry [12,14]  SEM [15]; PCR [17] |
| Histological analysis | Confocal microscopy [6] |
| Radiation exposure | Flow cytometry [7] |
| Cell quantification | Flow cytometry [16]  Light microscopy [1]  Phase-contrast microscopy [10] |
| Gene expression | PCR [13,15,19] |
| Enzymatic assay | Phase-contrast microscopy [10] |

SEM, scanning electron microscopy electron; PCR, polymerase chain reaction

1. Aleman J, George SK, Herberg S, Devarasetty M, Porada CD, Skardal A, et al. Deconstructed Microfluidic Bone Marrow On-A-Chip to Study Normal and Malignant Hemopoietic Cell–Niche Interactions. Small. 2019;15: 1–13. doi:10.1002/smll.201902971

2. Khin ZP, Ribeiro MLCC, Jacobson T, Hazlehurst L, Perez L, Baz R, et al. A preclinical assay for chemosensitivity in multiple myeloma. Cancer Res. 2014;74: 56–67. doi:10.1158/0008-5472.CAN-13-2397

3. Miller PGG, Shuler MLL. Design and demonstration of a pumpless 14 compartment microphysiological system. Biotechnol Bioeng. 2016;113: 2213–2227. doi:10.1002/bit.25989

4. Sung JH, Shuler ML. A micro cell culture analog (CCA) with 3-D hydrogel culture of multiple cell lines to assess metabolism-dependent cytotoxicity of anti-cancer drugs. Lab Chip. 2009;9: 1385–1394. doi:10.1039/b901377f

5. Sung JH, Kam C, Shuler ML. A microfluidic device for a pharmacokinetic-pharmacodynamic (PK-PD) model on a chip. Lab Chip. 2010;10: 446–455. doi:10.1039/b917763a

6. Torisawa YS, Spina CS, Mammoto T, Mammoto A, Weaver JC, Tat T, et al. Bone marrow-on-a-chip replicates hematopoietic niche physiology in vitro. Nat Methods. 2014;11: 663–669. doi:10.1038/nmeth.2938

7. Torisawa YS, Mammoto T, Jiang E, Jiang A, Mammoto A, Watters AL, et al. Modeling Hematopoiesis and Responses to Radiation Countermeasures in a Bone Marrow-on-a-Chip. Tissue Eng - Part C Methods. 2016;22: 509–515. doi:10.1089/ten.tec.2015.0507

8. Bruce A, Evans R, Mezan R, Shi L, Moses BS, Martin KH, et al. Three-dimensional microfluidic tri-culture model of the bone marrow microenvironment for study of acute lymphoblastic leukemia. PLoS One. 2015;10: 1–16. doi:10.1371/journal.pone.0140506

9. Zhang W, Gu Y, Sun Q, Siegel DS, Tolias P, Yang Z, et al. Ex vivo maintenance of primary human multiple myeloma cells through the optimization of the osteoblastic niche. PLoS One. 2015;10: 1–19. doi:10.1371/journal.pone.0125995

10. McAleer CW, Long CJ, Elbrecht D, Sasserath T, Bridges LR, Rumsey JW, et al. Multi-organ system for the evaluation of efficacy and off-target toxicity of anticancer therapeutics. Sci Transl Med. 2019;11. doi:10.1126/scitranslmed.aav1386

11. Houshmand M, Soleimani M, Atashi A, Saglio G, Abdollahi M, Zarif MN. Mimicking the Acute Myeloid Leukemia Niche for Molecular Study and Drug Screening. Tissue Eng - Part C Methods. 2017;23: 72–85. doi:10.1089/ten.tec.2016.0404

12. Thon JNN, Mazutis L, Wu S, Sylman JLL, Ehrlicher A, Machlus KRR, et al. Platelet bioreactor-on-a-chip. Blood. 2014;124: 1857–1867. doi:10.1182/blood-2014-05-574913

13. Marturano-Kruik A, Nava MM, Yeager K, Chramiec A, Hao L, Robinson S, et al. Human bone perivascular niche-on-a-chip for studying metastatic colonization. Proc Natl Acad Sci U S A. 2018;115: 1256–1261. doi:10.1073/pnas.1714282115

14. Chou DB, Frismantas V, Milton Y, David R, Pop-Damkov P, Ferguson D, et al. On-chip recapitulation of clinical bone marrow toxicities and patient-specific pathophysiology. Nat Biomed Eng. 2020;4: 394–406. doi:10.1038/s41551-019-0495-z

15. Sieber S, Wirth L, Cavak N, Koenigsmark M, Marx U, Lauster R, et al. Bone marrow-on-a-chip: Long-term culture of human haematopoietic stem cells in a three-dimensional microfluidic environment. J Tissue Eng Regen Med. 2018;12: 479–489. doi:10.1002/term.2507

16. Herland A, Maoz BM, Das D, Somayaji MR, Prantil-Baun R, Novak R, et al. Quantitative prediction of human pharmacokinetic responses to drugs via fluidically coupled vascularized organ chips. Nat Biomed Eng. 2020;4: 421–436. doi:10.1038/s41551-019-0498-9

17. Wuchter P, Saffrich R, Giselbrecht S, Nies C, Lorig H, Kolb S, et al. Microcavity arrays as an in vitro model system of the bone marrow niche for hematopoietic stem cells. Cell Tissue Res. 2016;364: 573–584. doi:10.1007/s00441-015-2348-8

18. Zhang W, Lee WY, Siegel DS, Tolias P, Zilberberg J. Patient-specific 3D microfluidic tissue model for multiple myeloma. Tissue Eng - Part C Methods. 2014;20: 663–670. doi:10.1089/ten.tec.2013.0490

19. Kotha SSS, Hayes BJJ, Phong KTT, Redd MAA, Bomsztyk K, Ramakrishnan A, et al. Engineering a multicellular vascular niche to model hematopoietic cell trafficking. Stem Cell Res Ther. 2018;9: 1–14. doi:10.1186/s13287-018-0808-2

20. Carrion B, Huang CPP, Ghajar CMM, Kachgal S, Kniazeva E, Jeon NLL, et al. Recreating the perivascular niche ex vivo using a microfluidic approach. Biotechnol Bioeng. 2010;107: 1020–1028. doi:10.1002/bit.22891

21. Zheng Y, Sun Y, Yu X, Shao Y, Zhang P, Dai G, et al. Angiogenesis in liquid tumors: An in-vitro assay for leukemic cell induced bone marrow angiogenesis. Adv Heal Mater. 2016;5: 1014–1024. doi:10.1016/j.physbeh.2017.03.040
