# Supplementary material for: Microfluidics and organ-on-a-chip technologies: A systematic review of the methods used to mimic bone marrow
Source: PLoS One. 2020 Dec 11;15(12):e0243840. doi: 10.1371/journal.pone.0243840 (PMC7732112; doi:10.1371/journal.pone.0243840)
Supplement: S1 Appendix — (DOCX) [file pone.0243840.s003.docx]

# S1 Appendix

This file details all the analyzed manuscripts as well as the exclusion criteria applied during the selection process. The selection process involved two steps which are detailed below.

**First step** – Publications were screened in their title and abstract content and those ones that did not contain the words "microfluidic" or "chip" combined with, "marrow", "hematopoietic", and "haematopoietic" were excluded (no microfluidic/OoC approach). After that, the remaining publications that did not report results or did not study any BM aspect were also excluded (irrelevant outcomes).

1. Peng XY, Li LQ, Hong HS. A vast-range speed control microchip for retention of all cell types. Lab Chip. 2009;9: 3012–3015. doi:10.1039/b818738j

2. Srisa-Art M, Bonzani IC, Williams A, Stevens MM, Demello AJ, Edel JB. Identification of rare progenitor cells from human periosteal tissue using droplet microfluidics. Analyst. 2009;134: 2239–2245. doi:10.1039/b910472k

3. Plouffe BD, Kniazeva T, Mayer JE, Murthy SK, Sales VL. Development of microfluidics as endothelial progenitor cell capture technology for cardiovascular tissue engineering and diagnostic medicine. FASEB J. 2009;23: 3309–3314. doi:10.1096/fj.09-130260

4. Markx GH, Carney L, Littlefair M, Sebastian A, Buckle AM. Recreating the hematon: Microfabrication of artificial haematopoietic stem cell microniches in vitro using dielectrophoresis. Biomed Microdevices. 2009;11: 143–150. doi:10.1007/s10544-008-9219-y

5. Tatosian DA, Shuler ML. A novel system for evaluation of drug mixtures for potential efficacy in treating multidrug resistant cancers. Biotechnol Bioeng. 2009;103: 187–198. doi:10.1002/bit.22219

6. Lin P, Chen L, Li D, Yang N, Sun Y, Xu Y. Dynamic analysis of bone marrow mesenchymal stem cells migrating to pancreatic islets using coculture microfluidic chips: An accelerated migrating rate and better survival of pancreatic islets were revealed. Neuroendocrinol Lett. 2009;30: 204–208.

7. Lautenschläger F, Paschke S, Schinkinger S, Bruel A, Beil M, Guck J. The regulatory role of cell mechanics for migration of differentiating myeloid cells. Proc Natl Acad Sci U S A. 2009;106: 15696–15701. doi:10.1073/pnas.0811261106

8. Wu HW, Hsu RC, Lin CC, Hwang SM, Lee G Bin. An integrated microfluidic system for isolation, counting, and sorting of hematopoietic stem cells. Biomicrofluidics. 2010;4. doi:10.1063/1.3454767

9. Seigneuric R, Markey L, S.A. Nuyten D, Dubernet C, T.A. Evelo C, Finot E, et al. From Nanotechnology to Nanomedicine: Applications to Cancer Research. Curr Mol Med. 2010;10: 640–652. doi:10.2174/156652410792630634

10. Schwertz H, Köster S, Kahr WHA, Michetti N, Kraemer BF, Weitz DA, et al. Anucleate platelets generate progeny. Blood. 2010;115: 3801–3809. doi:10.1182/blood-2009-08-239558

11. Petriv OI, Kuchenbauer F, Delaney AD, Lecault V, White A, Kent D, et al. Comprehensive microRNA expression profiling of the hematopoietic hierarchy. Proc Natl Acad Sci U S A. 2010;107: 15443–15448. doi:10.1073/pnas.1009320107

12. Gardinier JD, Townend CW, Jen KP, Wu Q, Duncan RL, Wang L. In situ permeability measurement of the mammalian lacunar-canalicular system. Bone. 2010;46: 1075–1081. doi:10.1016/j.bone.2010.01.371

13. Bauer M, Su G, Beebe DJ, Friedl A. 3D microchannel co-culture: Method and biological validation. Integr Biol. 2010;2: 371–378. doi:10.1039/c0ib00001a

14. Fang C, Wang Y, Vu NT, Lin WY, Hsieh Y Te, Rubbi L, et al. Integrated microfluidic and imaging platform for a kinase activity radioassay to analyze minute patient cancer samples. Cancer Res. 2010;70: 8299–8308. doi:10.1158/0008-5472.CAN-10-0851

15. Daud SS, Ibrahim K, Choong SS, Vengidasan L, Chong LA, Ariffin H. Microfluidic chip-based assay for post-hematopoietic stem cell transplantation chimerism monitoring using polymorphic tandem repeat markers. Anal Biochem. 2010;397: 181–185. doi:10.1016/j.ab.2009.10.008

16. Stott SL, Richard L, Nagrath S, Min Y, Miyamoto DT, Ulkus L, et al. Isolation and characterization of circulating tumor cells from patients with localized and metastatic prostate cancer. Sci Transl Med. 2010;2. doi:10.1126/scitranslmed.3000403

17. Mahara A, Yamaoka T. Antibody-immobilized column for quick cell separation based on cell rolling. Biotechnol Prog. 2010;26: 441–447. doi:10.1002/btpr.354

18. Luni C, Feldman HC, Pozzobon M, De Coppi P, Meinhart CD, Elvassore N. Microliter-bioreactor array with buoyancy-driven stirring for human hematopoietic stem cell culture. Biomicrofluidics. 2010;4. doi:10.1063/1.3380627

19. Zanardi A, Bandiera D, Bertolini F, Corsini CA, Gregato G, Milani P, et al. Miniaturized FISH for screening of onco-hematological malignancies. Biotechniques. 2010;49: 497–504. doi:10.2144/000113445

20. Markway BD, Tan GK, Brooke G, Hudson JE, Cooper-White JJ, Doran MR. Enhanced chondrogenic differentiation of human bone marrow-derived mesenchymal stem cells in low oxygen environment micropellet cultures. Cell Transplant. 2010;19: 29–42. doi:10.3727/096368909X478560

21. Saliba AE, Saias L, Psychari E, Minc N, Simon D, Bidard FC, et al. Microfluidic sorting and multimodal typing of cancer cells in self-assembled magnetic arrays. Proc Natl Acad Sci U S A. 2010;107: 14524–14529. doi:10.1073/pnas.1001515107

22. Vishnu VP, Lenferink A, Van Manen HJ, Subramaniam V, Van Blitterswijk CA, Otto C. Microbioreactors for raman microscopy of stromal cell differentiation. Anal Chem. 2010;82: 1844–1850. doi:10.1021/ac902515c

23. Carrier E, Srivastava A, de Necochea-Campion R, Takayama S, Yasukawa S, W. Basak G, et al. Artificial Hematopoietic Stem Cell Niche: Bioscaffolds to Microfluidics to Mathematical Simulations. Curr Top Med Chem. 2011;11: 1599–1605. doi:10.2174/156802611796117568

24. Mills RJ, Frith JE, Hudson JE, Cooper-White JJ. Effect of geometric challenges on cell migration. Tissue Eng - Part C Methods. 2011;17: 999–1010. doi:10.1089/ten.tec.2011.0138

25. Ulrike Haessler, Marco Pisano, Mingming Wu, Swartz MA. Dendritic cell chemotaxis in 3D under defined chemokine gradients reveals differential response to ligands CCL21 and CCL19. Proc Natl Acad Sci U S A. 2011;108: 5614–5619. doi:10.1073/pnas.1014920108

26. Adelaide Asnaghi M, Candiani G, Farè S, Fiore GB, Petrini P, Raimondi MT, et al. Trends in biomedical engineering: Focus on regenerative medicine. J Appl Biomater Biomech. 2011;9: 73–86. doi:10.5301/JABB.2011.8562

27. Kurkuri MD, Al-Ejeh F, Shi JY, Palms D, Prestidge C, Griesser HJ, et al. Plasma functionalized PDMS microfluidic chips: Towards point-of-care capture of circulating tumor cells. J Mater Chem. 2011;21: 8841–8848. doi:10.1039/c1jm10317b

28. Sekulovic S, Gasparetto M, Lecault V, Hoesli CA, Kent DG, Rosten P, et al. Ontogeny stage-independent and high-level clonal expansion in vitro of mouse hematopoietic stem cells stimulated by an engineered NUP98-HOX fusion transcription factor. Blood. 2011;118: 4366–4376. doi:10.1182/blood-2011-04-350066

29. Hubel A. Advancing the preservation of cellular therapy products. Transfusion. 2011;51: 82S-86S. doi:10.1111/j.1537-2995.2011.03370.x

30. Glotzbach JP, Januszyk M, Vial IN, Wong VW, Gelbard A, Kalisky T, et al. An information theoretic, microfluidic-based single cell analysis permits identification of subpopulations among putatively homogeneous stem cells. PLoS One. 2011;6. doi:10.1371/journal.pone.0021211

31. Su X, Qiu Y, Marquez-Curtis L, Gupta M, Capjack CE, Rozmus W, et al. Label-free and noninvasive optical detection of the distribution of nanometer-size mitochondria in single cells. J Biomed Opt. 2011;16: 067003. doi:10.1117/1.3583577

32. Widmer RP, Ferguson SJ. A mixed boundary representation to simulate the displacement of a biofluid by a biomaterial in porous media. J Biomech Eng. 2011;133. doi:10.1115/1.4003735

33. Gothard D, Tare RS, Mitchell PD, Dawson JI, Oreffo ROC. In search of the skeletal stem cell: Isolation and separation strategies at the macro/micro scale for skeletal regeneration. Lab Chip. 2011;11: 1206–1220. doi:10.1039/c0lc00575d

34. Greiner C, Hunter M, Rius F, Huang P, Georgakoudi I. Confocal backscattering-based detection of leukemic cells in flowing blood samples. Cytom Part A. 2011;79 A: 874–883. doi:10.1002/cyto.a.21086

35. Ricart BG, John B, Lee D, Hunter CA, Hammer DA. Dendritic Cells Distinguish Individual Chemokine Signals through CCR7 and CXCR4. J Immunol. 2011;186: 53–61. doi:10.4049/jimmunol.1002358

36. Dykes J, Lenshof A, Åstrand-Grundström IB, Laurell T, Scheding S. Efficient removal of platelets from peripheral blood progenitor cell products using a novel micro-chip based acoustophoretic platform. PLoS One. 2011;6. doi:10.1371/journal.pone.0023074

37. Lin H, Balic M, Zheng S, Datar R, Cote RJ. Disseminated and circulating tumor cells: Role in effective cancer management. Crit Rev Oncol Hematol. 2011;77: 1–11. doi:10.1016/j.critrevonc.2010.04.008

38. Heuzé ML, Collin O, Terriac E, Lennon-Duménil AM, Piel M. Cell migration in confinement: A micro-channel-based assay. Methods Mol Biol. 2011;769: 415–434. doi:10.1007/978-1-61779-207-6_28

39. Christophis C, Taubert I, Meseck GR, Schubert M, Grunze M, Ho AD, et al. Shear stress regulates adhesion and rolling of cd44+ leukemic and hematopoietic progenitor cells on hyaluronan. Biophys J. 2011;101: 585–593. doi:10.1016/j.bpj.2011.05.045

40. Lee WC, Bhagat AAS, Huang S, Van Vliet KJ, Han J, Lim CT. High-throughput cell cycle synchronization using inertial forces in spiral microchannels. Lab Chip. 2011;11: 1359–1367. doi:10.1039/c0lc00579g

41. Alves P, Pinto S, Kaiser JP, Bruinink A, de Sousa HC, Gil MH. Surface grafting of a thermoplastic polyurethane with methacrylic acid by previous plasma surface activation and by ultraviolet irradiation to reduce cell adhesion. Colloids Surfaces B Biointerfaces. 2011;82: 371–377. doi:10.1016/j.colsurfb.2010.09.021

42. Schirhagl R, Fuereder I, Hall EW, Medeiros BC, Zare RN. Microfluidic purification and analysis of hematopoietic stem cells from bone marrow. Lab Chip. 2011;11: 3130–3135. doi:10.1039/c1lc20353c

43. Ekpenyong AE, Whyte G, Chalut K, Pagliara S, Lautenschläger F, Fiddler C, et al. Viscoelastic Properties of Differentiating Blood Cells Are Fate- and Function-Dependent. PLoS One. 2012;7. doi:10.1371/journal.pone.0045237

44. Sun J, Gao Y, Isaacs RJ, Boelte KC, Charles Lin P, Boczko EM, et al. Simultaneous on-chip DC dielectrophoretic cell separation and quantitative separation performance characterization. Anal Chem. 2012;84: 2017–2024. doi:10.1021/ac203212g

45. Khan OF, Chamberlain MD, Sefton M V. Toward an in vitro vasculature: Differentiation of mesenchymal stromal cells within an endothelial cell-seeded modular construct in a microfluidic flow chamber. Tissue Eng - Part A. 2012;18: 744–756. doi:10.1089/ten.tea.2011.0058

46. Frith JE, Mills RJ, Hudson JE, Cooper-White JJ. Tailored integrin-extracellular matrix interactions to direct human mesenchymal stem cell differentiation. Stem Cells Dev. 2012;21: 2442–2456. doi:10.1089/scd.2011.0615

47. Hatch A, Pesko DM, Murthy SK. Tag-free microfluidic separation of cells against multiple markers. Anal Chem. 2012;84: 4618–4621. doi:10.1021/ac300496q

48. Abaci HE, Devendra R, Soman R, Drazer G, Gerecht S. Microbioreactors to manipulate oxygen tension and shear stress in the microenvironment of vascular stem and progenitor cells. Biotechnol Appl Biochem. 2012;59: 97–105. doi:10.1002/bab.1010

49. Jaramillo-Ferrada PA, Wolvetang EJ, Cooper-White JJ. Differential mesengenic potential and expression of stem cell-fate modulators in mesenchymal stromal cells from human-term placenta and bone marrow. J Cell Physiol. 2012;227: 3234–3242. doi:10.1002/jcp.24014

50. Plouffe BD, Mahalanabis M, Lewis LH, Klapperich CM, Murthy SK. Clinically relevant microfluidic magnetophoretic isolation of rare-cell populations for diagnostic and therapeutic monitoring applications. Anal Chem. 2012;84: 1336–1344. doi:10.1021/ac2022844

51. Smith MH. Cancer stem cell literatures. Stem Cell. 2012;3: 57–84. Available: https://www.scopus.com/inward/record.uri?eid=2-s2.0-84887853057&partnerID=40&md5=d8ac88fbaadc44aaea57ee58b8977854

52. Xu C, Poh YKC, Roes I, O’Cearbhaill ED, Matthiesen ME, Mu L, et al. A Portable Chemotaxis Platform for Short and Long Term Analysis. PLoS One. 2012;7: e44995. doi:10.1371/journal.pone.0044995

53. Wu L, Martin T, Li Y, Yang L, Halpenny M, Giulivi A, et al. Cell aggregation in thawed haematopoietic stem cell products visualised using micro-flow imaging. Transfusion Medicine. England; 2012. pp. 218–220. doi:10.1111/j.1365-3148.2012.01147.x

54. Galimberti S, Guerrini F, Salvi F, Petrini I, Gioia D, Messa E, et al. Arsenic trioxide and ascorbic acid interfere with the BCL2 family genes in patients with myelodysplastic syndromes: An ex-vivo study. J Hematol Oncol. 2012;5: 53. doi:10.1186/1756-8722-5-53

55. Ma Z, Liu Q, Liu H, Yang H, Yun JX, Eisenberg C, et al. Laser-patterned stem-cell bridges in a cardiac muscle model for on-chip electrical conductivity analyses. Lab Chip. 2012;12: 566–573. doi:10.1039/c2lc20699d

56. Lawrenz A, Nason F, Cooper-White JJ. Geometrical effects in microfluidic-based microarrays for rapid, efficient single-cell capture of mammalian stem cells and plant cells. Biomicrofluidics. 2012;6: 24112–2411217. doi:10.1063/1.4704521

57. Griessinger E, Jayasinghe SN, Bonnet D. Aerodynamically assisted bio-jetting of hematopoietic stem cells. Analyst. 2012;137: 1329–1333. doi:10.1039/c2an16027g

58. Wildenhain S, Ingenhag D, Ruckert C, Degistirici Ö, Dugas M, Meisel R, et al. Homeobox protein HB9 binds to the prostaglandin E receptor 2 promoter and inhibits intracellular cAMP mobilization in leukemic cells. J Biol Chem. 2012;287: 40703–40712. doi:10.1074/jbc.M111.308965

59. Kobel SA, Burri O, Griffa A, Girotra M, Seitz A, Lutolf MP. Automated analysis of single stem cells in microfluidic traps. Lab Chip. 2012;12: 2843–2849. doi:10.1039/c2lc40317j

60. Warrick JW, Young EWK, Schmuck EG, Saupe KW, Beebe DJ. High-content adhesion assay to address limited cell samples. Integr Biol (United Kingdom). 2013;5: 720–727. doi:10.1039/c3ib20224k

61. Cuthbert RJ, Churchman SM, Tan HB, McGonagle D, Jones E, Giannoudis P V. Induced periosteum a complex cellular scaffold for the treatment of large bone defects. Bone. 2013;57: 484–492. doi:10.1016/j.bone.2013.08.009

62. Shamloo A, Manchandia M, Mani M, Mani M, Nguyen C, Jahn T, et al. Complex chemoattractive and chemorepellent Kit signals revealed by direct imaging of murine mast cells in microfluidic gradient chambers. Integr Biol (United Kingdom). 2013;5: 1076–1085. doi:10.1039/c3ib40025e

63. Marker DF, Tremblay MÈ, Puccini JM, Barbieri J, Gantz Marker MA, Loweth CJ, et al. The new small-molecule mixed-lineage kinase 3 inhibitor URMC-099 is neuroprotective and anti-inflammatory in models of human immunodeficiency virus-associated neurocognitive disorders. J Neurosci. 2013;33: 9998–10010. doi:10.1523/JNEUROSCI.0598-13.2013

64. Marx V. Where stem cells call home. Nat Methods. 2013;10: 111–115. doi:10.1038/nmeth.2336

65. Zanardi A, Barborini E, Carbone R. MicroFIND β Approach to Fluorescent in Situ Hybridization (FISH). Methods Mol Biol. 2013;949: 433–449. doi:10.1007/978-1-62703-134-9_28

66. Huang HC, Chang YJ, Chen WC, Harn HIC, Tang MJ, Wu CC. Enhancement of renal epithelial cell functions through microfluidic-based coculture with adipose-derived stem cells. Tissue Eng - Part A. 2013;19: 2024–2034. doi:10.1089/ten.tea.2012.0605

67. Mohanty JG, Nagababu E, Friedman JS, Rifkind JM. SOD2 deficiency in hematopoietic cells in mice results in reduced red blood cell deformability and increased heme degradation. Exp Hematol. 2013;41: 316–321. doi:10.1016/j.exphem.2012.10.017

68. Alshareef M, Metrakos N, Juarez Perez E, Azer F, Yang F, Yang X, et al. Separation of tumor cells with dielectrophoresis-based microfluidic chip. Biomicrofluidics. 2013;7. doi:10.1063/1.4774312

69. Salerno A, Levato R, Mateos-Timoneda MA, Engel E, Netti PA, Planell JA. Modular polylactic acid microparticle-based scaffolds prepared via microfluidic emulsion/solvent displacement process: Fabrication, characterization, and in vitro mesenchymal stem cells interaction study. J Biomed Mater Res - Part A. 2013;101 A: 720–732. doi:10.1002/jbm.a.34374

70. Zhang X, Zhang C, Li Z, Zhong J, Weiner LP, Zhong JF. Investigating evolutionary perspective of carcinogenesis with single-cell transcriptome analysis. Chin J Cancer. 2013;32: 636–639. doi:10.5732/cjc.012.10291

71. Mendelson A, Cheung YK, Paluch K, Chen M, Kong K, Tan J, et al. Competitive stem cell recruitment by multiple cytotactic cues. Lab Chip. 2013;13: 1156–1164. doi:10.1039/c2lc41219e

72. Moonsamy P V., Williams T, Bonella P, Holcomb CL, Höglund BN, Hillman G, et al. High throughput HLA genotyping using 454 sequencing and the Fluidigm Access ArrayTM system for simplified amplicon library preparation. Tissue Antigens. 2013;81: 141–149. doi:10.1111/tan.12071

73. Lozito TP, Alexander PG, Lin H, Gottardi R, Cheng AWM, Tuan RS. Three-dimensional osteochondral microtissue to model pathogenesis of osteoarthritis. Stem Cell Res Ther. 2013;4: S6. doi:10.1186/scrt367

74. Kuddannaya S, Chuah YJ, Lee MHA, Menon N V., Kang Y, Zhang Y. Surface chemical modification of poly(dimethylsiloxane) for the enhanced adhesion and proliferation of mesenchymal stem cells. ACS Appl Mater Interfaces. 2013;5: 9777–9784. doi:10.1021/am402903e

75. Sahoo MK, Lefterova MI, Yamamoto F, Waggoner JJ, Chou S, Holmes SP, et al. Detection of cytomegalovirus drug resistance mutations by next-Generation sequencing. J Clin Microbiol. 2013;51: 3700–3710. doi:10.1128/JCM.01605-13

76. Krishnamurthy S, Bischoff F, Ann Mayer J, Wong K, Pham T, Kuerer H, et al. Discordance in HER2 gene amplification in circulating and disseminated tumor cells in patients with operable breast cancer . Cancer Med. 2013;2: 226–233. doi:10.1002/cam4.70

77. Konry T, Golberg A, Yarmush M. Live single cell functional phenotyping in droplet nano-liter reactors. Sci Rep. 2013;3: 3179. doi:10.1038/srep03179

78. Titmarsh DM, Chen H, Wolvetang EJ, Cooper-White JJ. Arrayed cellular environments for stem cells and regenerative medicine. Biotechnol J. 2013;8: 167–179. doi:10.1002/biot.201200149

79. Su HW, Prieto JL, Voldman J. Rapid dielectrophoretic characterization of single cells using the dielectrophoretic spring. Lab Chip. 2013;13: 4109–4117. doi:10.1039/c3lc50392e

80. Mach AJ, Adeyiga OB, Di Carlo D. Microfluidic sample preparation for diagnostic cytopathology. Lab Chip. 2013;13: 1011–1026. doi:10.1039/c2lc41104k

81. Sheng W, Chen T, Tan W, Fan ZH. Multivalent DNA nanospheres for enhanced capture of cancer cells in microfluidic devices. ACS Nano. 2013;7: 7067–7076. doi:10.1021/nn4023747

82. Matern D, Oglesbee D, Tortorelli S. Newborn screening for lysosomal storage disorders and other neuronopathic conditions. Dev Disabil Res Rev. 2013;17: 247–253. doi:10.1002/ddrr.1117

83. Dudani JS, Gossett DR, Tse HTK, Di Carlo D. Pinched-flow hydrodynamic stretching of single-cells. Lab Chip. 2013;13: 3728–3734. doi:10.1039/c3lc50649e

84. Yu W, Qu H, Hu G, Zhang Q, Song K, Guan H, et al. A microfluidic-based multi-shear device for investigating the effects of low fluid-induced stresses on osteoblasts. PLoS One. 2014;9. doi:10.1371/journal.pone.0089966

85. Mclaughlin LM, Xu H, Carden SE, Fisher S, Reyes M, Heilshorn SC, et al. A microfluidic-based genetic screen to identify microbial virulence factors that inhibit dendritic cell migration. Integr Biol (United Kingdom). 2014;6: 438–449. doi:10.1039/c3ib40177d

86. Khaldoyanidi SK, Goncharova V, Mueller B, Schraufstatter IU. Hyaluronan in the healthy and malignant hematopoietic microenvironment. Adv Cancer Res. 2014;123: 149–189. doi:10.1016/B978-0-12-800092-2.00006-X

87. Bischel LL, Casavant BP, Young PA, Eliceiri KW, Basu HS, Beebe DJ. A microfluidic coculture and multiphoton FAD analysis assay provides insight into the influence of the bone microenvironment on prostate cancer cells. Integr Biol (United Kingdom). 2014;6: 627–635. doi:10.1039/c3ib40240a

88. Berry SM, Singh C, Lang JD, Strotman LN, Alarid ET, Beebe DJ. Streamlining gene expression analysis: Integration of co-culture and mRNA purification. Integr Biol (United Kingdom). 2014;6: 224–231. doi:10.1039/c3ib40136g

89. Chandrasekaran S, McGuire MJ, King MR. Sweeping lymph node micrometastases off their feet: An engineered model to evaluate natural killer cell mediated therapeutic intervention of circulating tumor cells that disseminate to the lymph nodes. Lab Chip. 2014;14: 118–127. doi:10.1039/c3lc50584g

90. Hanke M, Hoffmann I, Christophis C, Schubert M, Hoang VT, Zepeda-Moreno A, et al. Differences between healthy hematopoietic progenitors and leukemia cells with respect to CD44 mediated rolling versus adherence behavior on hyaluronic acid coated surfaces. Biomaterials. 2014;35: 1411–1419. doi:10.1016/j.biomaterials.2013.11.011

91. Mahmood S, Nandagopal S, Sow I, Lin F, Kung SKP. Microfluidic-based, live-cell analysis allows assessment of NK-cell migration in response to crosstalk with dendritic cells. Eur J Immunol. 2014;44: 2737–2748. doi:10.1002/eji.201344244

92. Hedges EA, Hughes AD, Liesveld JL, King MR. Modulation of Selectin-Mediated Adhesion of Flowing Lymphoma and Bone Marrow Cells by Immobilized SDF-1. Int J Mol Sci. 2014;15: 15061–15072. doi:10.3390/ijms150915061

93. Lenshof A, Jamal A, Dykes J, Urbansky A, Åstrand-Grundström I, Laurell T, et al. Efficient purification of CD4+ lymphocytes from peripheral blood progenitor cell products using affinity bead acoustophoresis. Cytom Part A. 2014;85: 933–941. doi:10.1002/cyto.a.22507

94. Witters D, Sun B, Begolo S, Rodriguez-Manzano J, Robles W, Ismagilov RF. Digital biology and chemistry. Lab Chip. 2014;14: 3225–3232. doi:10.1039/c4lc00248b

95. Shu Z, Heimfeld S, Gao D. Hematopoietic SCT with cryopreserved grafts: Adverse reactions after transplantation and cryoprotectant removal before infusion. Bone Marrow Transplant. 2014;49: 469–476. doi:10.1038/bmt.2013.152

96. Shalek AK, Satija R, Shuga J, Trombetta JJ, Gennert D, Lu D, et al. Single-cell RNA-seq reveals dynamic paracrine control of cellular variation. Nature. 2014;510: 363–369. doi:10.1038/nature13437

97. Carpenter EL, Rader JA, Ruden J, Rappaport EF, Hunter KN, Hallberg PL, et al. Dielectrophoretic capture and genetic analysis of single neuroblastoma tumor cells. Front Oncol. 2014;4 JUL: 201. doi:10.3389/fonc.2014.00201

98. Patel V, Chen LS, Wierda WG, Balakrishnan K, Gandhi V. Impact of bone marrow stromal cells on Bcl-2 family members in chronic lymphocytic leukemia. Leuk Lymphoma. 2014;55: 899–910. doi:10.3109/10428194.2013.819573

99. Heikal N, Nussenzveig RH, Agarwal AM. Deparaffinization with mineral oil: A simple procedure for extraction of high-quality DNA from archival formalin-fixed paraffin-embedded samples. Appl Immunohistochem Mol Morphol. 2014;22: 623–626. doi:10.1097/PAI.0b013e3182a77bfe

100. Lee WC, Shi H, Poon Z, Nyan LM, Kaushik T, Shivashankar G V., et al. Multivariate biophysical markers predictive of mesenchymal stromal cell multipotency. Proc Natl Acad Sci U S A. 2014;111: E4409–E4418. doi:10.1073/pnas.1402306111

101. Hu W, Park CY. Measuring MicroRNA expression in mouse hematopoietic stem cells. Methods Mol Biol. 2014;1185: 121–140. doi:10.1007/978-1-4939-1133-2_8

102. Wang CH, Lee YH, Kuo HT, Liang WF, Li WJ, Lee G Bin. Dielectrophoretically-assisted electroporation using light-activated virtual microelectrodes for multiple DNA transfection. Lab Chip. 2014;14: 592–601. doi:10.1039/c3lc51102b

103. Mahara A, Chen H, Ishihara K, Yamaoka T. Phospholipid polymer-based antibody immobilization for cell rolling surfaces in stem cell purification system. J Biomater Sci Polym Ed. 2014;25: 1590–1601. doi:10.1080/09205063.2014.936926

104. Wu Y, Wang L, Guo B, Ma PX. Injectable biodegradable hydrogels and microgels based on methacrylated poly(ethylene glycol)-co-poly(glycerol sebacate) multi-block copolymers: Synthesis, characterization, and cell encapsulation. J Mater Chem B. 2014;2: 3674–3685. doi:10.1039/c3tb21716g

105. Koltsova EK, Sundd P, Zarpellon A, Ouyang H, Mikulski Z, Zampolli A, et al. Genetic deletion of platelet glycoprotein lb alpha but not its extracellular domain protects from atherosclerosis. Thromb Haemost. 2014;112: 1252–1263. doi:10.1160/TH14-02-0130

106. Almeida M, García-Montero AC, Orfao A. Cell purification: A new challenge for biobanks. Pathobiology. 2014;81: 261–275. doi:10.1159/000358306

107. Lange V, Böhme I, Hofmann J, Lang K, Sauter J, Schöne B, et al. Cost-efficient high-throughput HLA typing by MiSeq amplicon sequencing. BMC Genomics. 2014;15: 63. doi:10.1186/1471-2164-15-63

108. Koepsell SA, Burright-Hittner K, Landmark JD. Evans syndrome in a pediatric liver transplant recipient with an autoantibody with apparent specificity for the KEL4 (Kpb) antigen. Immunohematology. 2014;30: 14–17. Available: https://www.scopus.com/inward/record.uri?eid=2-s2.0-84908051545&partnerID=40&md5=a1ba7b3cd11c1067e8828b3e4c0f4cdb

109. Ding X, Shagoshtasbi H, Deng P, Lee YK. A Nonlinear Size-Dependent Equivalent Circuit Model for Single-Cell Electroporation on Microfluidic Chips. J Lab Autom. 2015;20: 481–490. doi:10.1177/2211068215573189

110. Sekhavati F, Endele M, Rappl S, Marel AK, Schroeder T, Rädler JO. Marker-free detection of progenitor cell differentiation by analysis of Brownian motion in micro-wells. Integr Biol (United Kingdom). 2015;7: 178–183. doi:10.1039/c4ib00158c

111. Huang S Bin, Chou D, Chang YH, Li KC, Chiu TK, Ventikos Y, et al. Development of a pneumatically driven active cover lid for multi-well microplates for use in perfusion three-dimensional cell culture. Sci Rep. 2015;5: 1–14. doi:10.1038/srep18352

112. Li N, Diaz MF, Wenzel PL. Application of fluid mechanical force to embryonic sources of hemogenic endothelium and hematopoietic stem cells. Methods Mol Biol. 2015;1212: 183–193. doi:10.1007/7651_2014_95

113. Lewis DM, Abaci HE, Xu Y, Gerecht S. Endothelial progenitor cell recruitment in a microfluidic vascular model. Biofabrication. 2015;7. doi:10.1088/1758-5090/7/4/045010

114. Caliari SR, Gonnerman EA, Grier WK, Weisgerber DW, Banks JM, Alsop AJ, et al. Collagen scaffold arrays for combinatorial screening of biophysical and biochemical regulators of cell behavior. Adv Healthc Mater. 2015;4: 58–64. doi:10.1002/adhm.201400252

115. Cao Z, Chen C, He B, Tan K, Lu C. A microfluidic device for epigenomic profiling using 100 cells. Nat Methods. 2015;12: 959–962. doi:10.1038/nmeth.3488

116. Banks TA, Luckman PSB, Frith JE, Cooper-White JJ. Effects of electric fields on human mesenchymal stem cell behaviour and morphology using a novel multichannel device. Integr Biol (United Kingdom). 2015;7: 693–712. doi:10.1039/c4ib00297k

117. Pagella P, Neto E, Lamghari M, Mitsiadis TA. Investigation of orofacial stem cell niches and their innervation through microfluidic devices. Eur Cells Mater. 2015;29: 213–223. doi:10.22203/eCM.v029a16

118. Jeon JS, Bersini S, Gilardi M, Dubini G, Charest JL, Moretti M, et al. Human 3D vascularized organotypic microfluidic assays to study breast cancer cell extravasation. Proc Natl Acad Sci U S A. 2015;112: 214–219. doi:10.1073/pnas.1417115112

119. Rath S, Salinas M, Bhatacharjee S, Ramaswamy S. Marrow stem cell differentiation for valvulogenesis via oscillatory flow and nicotine agonists: Unusual suspects? J Long Term Eff Med Implants. 2015;25: 147–160. doi:10.1615/JLongTermEffMedImplants.2015011695

120. Li S, Plouffe BD, Belov AM, Ray S, Wang X, Murthy SK, et al. An integrated platform for isolation, processing, and mass spectrometry-based proteomic profiling of rare cells in whole blood. Mol Cell Proteomics. 2015;14: 1672–1683. doi:10.1074/mcp.M114.045724

121. Kim J, Lee H, Selimović Š, Gauvin R, Bae H. Organ-On-A-Chip: Development and Clinical Prospects Toward Toxicity Assessment with an Emphasis on Bone Marrow. Drug Saf. 2015;38: 409–418. doi:10.1007/s40264-015-0284-x

122. Zhou B, Gao X, Wang C, Ye Z, Gao Y, Xie J, et al. Functionalized PDMS with Versatile and Scalable Surface Roughness Gradients for Cell Culture. ACS Appl Mater Interfaces. 2015;7: 17181–17187. doi:10.1021/acsami.5b04032

123. Rotem A, Ram O, Shoresh N, Sperling RA, Goren A, Weitz DA, et al. Single-cell ChIP-seq reveals cell subpopulations defined by chromatin state. Nat Biotechnol. 2015;33: 1165–1172. doi:10.1038/nbt.3383

124. Song H, Rosano JM, Wang Y, Garson CJ, Prabhakarpandian B, Pant K, et al. Continuous-flow sorting of stem cells and differentiation products based on dielectrophoresis. Lab Chip. 2015;15: 1320–1328. doi:10.1039/c4lc01253d

125. Otto O, Rosendahl P, Mietke A, Golfier S, Herold C, Klaue D, et al. Real-time deformability cytometry: On-the-fly cell mechanical phenotyping. Nat Methods. 2015;12: 199–202. doi:10.1038/nmeth.3281

126. Chabaud M, Heuze ML, Bretou M, Vargas P, Maiuri P, Solanes P, et al. Cell migration and antigen capture are antagonistic processes coupled by myosin II in dendritic cells. Nat Commun. 2015;6: 7526. doi:10.1038/ncomms8526

127. Jung H, Chun MS, Chang MS. Sorting of human mesenchymal stem cells by applying optimally designed microfluidic chip filtration. Analyst. 2015;140: 1265–1274. doi:10.1039/c4an01430h

128. Kamat V, Muthard RW, Li R, Diamond SL. Microfluidic assessment of functional culture-derived platelets in human thrombi under flow. Exp Hematol. 2015;43: 891-900.e4. doi:10.1016/j.exphem.2015.06.302

129. Benam KH, Dauth S, Hassell B, Herland A, Jain A, Jang K-J, et al. Engineered In Vitro Disease Models. Annu Rev Pathol Mech Dis. 2015;10: 195–262. doi:10.1146/annurev-pathol-012414-040418

130. Rommelaere S, Millet V, Rihet P, Atwell S, Helfer E, Chasson L, et al. Serum Pantetheinase/Vanin Levels regulate erythrocyte homeostasis and severity of malaria. Am J Pathol. 2015;185: 3039–3052. doi:10.1016/j.ajpath.2015.07.011

131. Heath DE, Sharif ARM, Ng CP, Rhoads MG, Griffith LG, Hammond PT, et al. Regenerating the cell resistance of micromolded PEG hydrogels. Lab Chip. 2015;15: 2073–2089. doi:10.1039/c4lc01416b

132. Pak C, Callander NS, Young EWK, Titz B, Kim KM, Saha S, et al. MicroC3: an ex vivo microfluidic cis-coculture assay to test chemosensitivity and resistance of patient multiple myeloma cells. Integr Biol (United Kingdom). 2015;7: 643–654. doi:10.1039/c5ib00071h

133. Vacchelli E, Ma Y, Baracco EE, Sistigu A, Enot DP, Pietrocola F, et al. Chemotherapy-induced antitumor immunity requires formyl peptide receptor 1. Science (80- ). 2015;350: 972–978. doi:10.1126/science.aad0779

134. Balakrishnan KR, Whang JC, Hwang R, Hack JH, Godley LA, Sohn LL. Node-pore sensing enables label-free surface-marker profiling of single cells. Anal Chem. 2015;87: 2988–2995. doi:10.1021/ac504613b

135. Cunha-Matos CA, Millington OR, Wark AW, Zagnoni M. Real-time assessment of nanoparticle-mediated antigen delivery and cell response. Lab Chip. 2016;16: 3374–3381. doi:10.1039/c6lc00599c

136. Anandi P, Tian X, Chinian F, Cantilena CR, Dunavin N, Hensel N, et al. Improved reproducibility and quality of GvHD biomarker assay: Application of multiplex microfluidic channel system. Bone Marrow Transplant. 2016;51: 1615–1616. doi:10.1038/bmt.2016.226

137. Lee D, Hwang B, Choi Y, Kim B. Negative dielectrophoretic force based cell sorter with simplified structure for high reliability. Int J Precis Eng Manuf. 2016;17: 247–251. doi:10.1007/s12541-016-0032-x

138. Xavier M, Oreffo ROC, Morgan H. Skeletal stem cell isolation: A review on the state-of-the-art microfluidic label-free sorting techniques. Biotechnol Adv. 2016;34: 908–923. doi:10.1016/j.biotechadv.2016.05.008

139. Nandagopal S, Lin F, Kung SKP. Microfluidic-based live-cell analysis of NK cell migration in vitro. Methods Mol Biol. 2016;1441: 75–86. doi:10.1007/978-1-4939-3684-7_7

140. Sugimura R. Bioengineering Hematopoietic Stem Cell Niche toward Regenerative Medicine. Adv Drug Deliv Rev. 2016;99: 212–220. doi:10.1016/j.addr.2015.10.010

141. de Seny D, Cobraiville G, Leprince P, Fillet M, Collin C, Mathieu M, et al. Biomarkers of inflammation and innate immunity in atrophic nonunion fracture. J Transl Med. 2016;14. doi:10.1186/s12967-016-1019-1

142. Mondal N, Stolfa G, Antonopoulos A, Zhu Y, Wang SS, Buffone A, et al. Glycosphingolipids on Human Myeloid Cells Stabilize E-Selectin-Dependent Rolling in the Multistep Leukocyte Adhesion Cascade. Arterioscler Thromb Vasc Biol. 2016;36: 718–727. doi:10.1161/ATVBAHA.115.306748

143. Wong QY, Liu N, Koh CG, Li HY, Lew WS. Isolation of magnetically tagged cancer cells through an integrated magnetofluidic device. Microfluid Nanofluidics. 2016;20. doi:10.1007/s10404-016-1804-4

144. Shahini A, Xia J, Zhou Z, Zhao Y, Cheng MMC. Versatile Miniature Tunable Liquid Lenses Using Transparent Graphene Electrodes. Langmuir. 2016;32: 1658–1665. doi:10.1021/acs.langmuir.5b03407

145. Ishii A, Ariyasu K, Mitsuhashi T, Heinemann D, Heisterkamp A, Terakawa M. Biodegradable microsphere-mediated cell perforation in microfluidic channel using femtosecond laser. J Biomed Opt. 2016;21: 055001. doi:10.1117/1.jbo.21.5.055001

146. Song H, Rosano JM, Wang Y, Garson CJ, Prabhakarpandian B, Pant K, et al. Identification of mesenchymal stem cell differentiation state using dual-micropore microfluidic impedance flow cytometry. Anal Methods. 2016;8: 7437–7444. doi:10.1039/c6ay01377e

147. Adeniran-Catlett AE, Weinstock LD, Bozal FK, Beguin E, Caraballo AT, Murthy SK. Accelerated adipogenic differentiation of hMSCs in a microfluidic shear stimulation platform. Biotechnol Prog. 2016;32: 440–446. doi:10.1002/btpr.2211

148. Marki A, Gutierrez E, Mikulski Z, Groisman A, Ley K. Microfluidics-based side view flow chamber reveals tether-to-sling transition in rolling neutrophils. Sci Rep. 2016;6: 28870. doi:10.1038/srep28870

149. Kashaninejad N, Nikmaneshi MR, Moghadas H, Oskouei AK, Rismanian M, Barisam M, et al. Organ-tumor-on-a-chip for chemosensitivity assay: A critical review. Micromachines. 2016;7. doi:10.3390/mi7080130

150. Fay ME, Myers DR, Kumar A, Turbyfield CT, Byler R, Crawford K, et al. Cellular softening mediates leukocyte demargination and trafficking, thereby increasing clinical blood counts. Proc Natl Acad Sci U S A. 2016;113: 1987–1992. doi:10.1073/pnas.1508920113

151. Kang KJ, Ju SM, Jang YJ, Kim J. Indirect co-culture of stem cells from human exfoliated deciduous teeth and oral cells in a microfluidic platform. Tissue Eng Regen Med. 2016;13: 428–436. doi:10.1007/s13770-016-0005-2

152. Xavier M, Rosendahl P, Herbig M, Kräter M, Spencer D, Bornhäuser M, et al. Mechanical phenotyping of primary human skeletal stem cells in heterogeneous populations by real-time deformability cytometry. Integr Biol (United Kingdom). 2016;8: 616–623. doi:10.1039/c5ib00304k

153. Bhuvanendran Nair Gourikutty S, Chang CP, Poenar DP. An integrated on-chip platform for negative enrichment of tumour cells. J Chromatogr B Anal Technol Biomed Life Sci. 2016;1028: 153–164. doi:10.1016/j.jchromb.2016.06.016

154. De Groot TE, Veserat KS, Berthier E, Beebe DJ, Theberge AB. Surface-tension driven open microfluidic platform for hanging droplet culture. Lab Chip. 2016;16: 334–344. doi:10.1039/c5lc01353d

155. Qian JY, Chopp M, Liu Z. Mesenchymal stromal cells promote axonal outgrowth alone and synergistically with astrocytes via tPA. PLoS One. 2016;11. doi:10.1371/journal.pone.0168345

156. Alhasan L, Qi A, Rezk AR, Yeo LY, Chan PPY. Assessment of the potential of a high frequency acoustomicrofluidic nebulisation platform for inhaled stem cell therapy. Integr Biol (United Kingdom). 2016;8: 12–20. doi:10.1039/c5ib00206k

157. Dumbleton J, Agarwal P, Huang H, Hogrebe N, Han R, Gooch KJ, et al. The Effect of RGD Peptide on 2D and Miniaturized 3D Culture of HEPM Cells, MSCs, and ADSCs with Alginate Hydrogel. Cell Mol Bioeng. 2016;9: 277–288. doi:10.1007/s12195-016-0428-9

158. Jackson JM, Taylor JB, Witek MA, Hunsucker SA, Waugh JP, Fedoriw Y, et al. Microfluidics for the detection of minimal residual disease in acute myeloid leukemia patients using circulating leukemic cells selected from blood. Analyst. 2016;141: 640–651. doi:10.1039/c5an01836f

159. McWhorter FY, Smith TD, Luu TU, Rahim MK, Haun JB, Liu WF. Macrophage secretion heterogeneity in engineered microenvironments revealed using a microwell platform. Integr Biol (United Kingdom). 2016;8: 751–760. doi:10.1039/c6ib00053c

160. Hvichia GE, Parveen Z, Wagner C, Janning M, Quidde J, Stein A, et al. A novel microfluidic platform for size and deformability based separation and the subsequent molecular characterization of viable circulating tumor cells. Int J Cancer. 2016;138: 2894–2904. doi:10.1002/ijc.30007

161. Zhao X, Liu S, Yildirimer L, Zhao H, Ding R, Wang H, et al. Injectable Stem Cell-Laden Photocrosslinkable Microspheres Fabricated Using Microfluidics for Rapid Generation of Osteogenic Tissue Constructs. Adv Funct Mater. 2016;26: 2809–2819. doi:10.1002/adfm.201504943

162. Fan Z, McArdle S, Marki A, Mikulski Z, Gutierrez E, Engelhardt B, et al. Neutrophil recruitment limited by high-affinity bent β2 integrin binding ligand in cis. Nat Commun. 2016;7: 12658. doi:10.1038/ncomms12658

163. Salamanna F, Contartese D, Maglio M, Fini M. A systematic review on in vitro 3d bone metastases models. A new horizon to recapitulate the native clinical scenario? Oncotarget. 2016;7: 44803–44820. doi:10.18632/oncotarget.8394

164. Albritton JL, Roybal JD, Paulsen SJ, Calafat NJ, Flores-Zaher JA, Farach-Carson MC, et al. Ultrahigh-throughput generation and characterization of cellular aggregates in laser-ablated microwells of poly(dimethylsiloxane). RSC Adv. 2016;6: 8980–8991. doi:10.1039/c5ra26022a

165. Liu F, Kc P, Zhang G, Zhe J. In situ single cell detection via microfluidic magnetic bead assay. PLoS One. 2017;12. doi:10.1371/journal.pone.0172697

166. Lucarini V, Buccione C, Ziccheddu G, Peschiaroli F, Sestili P, Puglisi R, et al. Combining Type I Interferons and 5-Aza-2′-Deoxycitidine to Improve Anti-Tumor Response against Melanoma. J Invest Dermatol. 2017;137: 159–169. doi:10.1016/j.jid.2016.08.024

167. Li W, Zhang Y, Reynolds CP, Pappas D. Microfluidic Separation of Lymphoblasts for the Isolation of Acute Lymphoblastic Leukemia Using the Human Transferrin Receptor as a Capture Target. Anal Chem. 2017;89: 7340–7347. doi:10.1021/acs.analchem.7b00377

168. Peterson VM, Zhang KX, Kumar N, Wong J, Li L, Wilson DC, et al. Multiplexed quantification of proteins and transcripts in single cells. Nat Biotechnol. 2017;35: 936–939. doi:10.1038/nbt.3973

169. Moore TA, Brodersen P, Young EWK. Multiple Myeloma Cell Drug Responses Differ in Thermoplastic vs PDMS Microfluidic Devices. Anal Chem. 2017;89: 11391–11398. doi:10.1021/acs.analchem.7b02351

170. Knapp DJHF, Hammond CA, Miller PH, Rabu GM, Beer PA, Ricicova M, et al. Dissociation of Survival, Proliferation, and State Control in Human Hematopoietic Stem Cells. Stem Cell Reports. 2017;8: 152–162. doi:10.1016/j.stemcr.2016.12.003

171. Li R, Hebert JD, Lee TA, Xing H, Boussommier-Calleja A, Hynes RO, et al. Macrophage-secreted TNFα and TGFβ1 influence migration speed and persistence of cancer cells in 3D tissue culture via independent pathways. Cancer Res. 2017;77: 279–290. doi:10.1158/0008-5472.CAN-16-0442

172. Xu H, Li Z, Guo Y, Peng X, Qin J. Probing the response of lung tumor cells to inflammatory microvascular endothelial cells on fluidic microdevice. Electrophoresis. 2017;38: 311–319. doi:10.1002/elps.201600278

173. Martinez AF, McMahon RD, Horner M, Miller WM. A uniform-shear rate microfluidic bioreactor for real-time study of proplatelet formation and rapidly-released platelets. Biotechnol Prog. 2017;33: 1614–1629. doi:10.1002/btpr.2563

174. Diaz M, Evans S, Olson S, Cox C, Wenzel P. A Co-culture Assay to Determine Efficacy of TNF-α Suppression by Biomechanically Induced Human Bone Marrow Mesenchymal Stem Cells. Bio-Protocol. 2017;7. doi:10.21769/bioprotoc.2513

175. Shah SB, Singh A. Creating artificial lymphoid tissues to study immunity and hematological malignancies. Curr Opin Hematol. 2017;24: 377–383. doi:10.1097/MOH.0000000000000356

176. Qasaimeh MA, Wu YC, Bose S, Menachery A, Talluri S, Gonzalez G, et al. Isolation of Circulating Plasma Cells in Multiple Myeloma Using CD138 Antibody-Based Capture in a Microfluidic Device. Sci Rep. 2017;7: 45681. doi:10.1038/srep45681

177. Xavier M, De Andres MC, Spencer D, Oreffo ROC, Morgan H. Size and dielectric properties of skeletal stem cells change critically after enrichment and expansion from human bone marrow: Consequences for microfluidic cell sorting. J R Soc Interface. 2017;14. doi:10.1098/rsif.2017.0233

178. Zhang W, Lee WY, Zilberberg J. Tissue engineering platforms to replicate the tumor microenvironment of multiple myeloma. Methods Mol Biol. 2017;1513: 171–191. doi:10.1007/978-1-4939-6539-7_12

179. Huang X, Farooq U, Chen J, Ge Y, Gao H, Su J, et al. A Surface Acoustic Wave Pumped Lensless Microfluidic Imaging System for Flowing Cell Detection and Counting. IEEE Trans Biomed Circuits Syst. 2017;11: 1478–1487. doi:10.1109/TBCAS.2017.2732828

180. Yildizhan Y, Erdem N, Islam M, Martinez-Duarte R, Elitas M. Dielectrophoretic separation of live and dead monocytes using 3D carbon-electrodes. Sensors (Switzerland). 2017;17. doi:10.3390/s17112691

181. Petrie Aronin CE, Zhao YM, Yoon JS, Morgan NY, Prüstel T, Germain RN, et al. Migrating Myeloid Cells Sense Temporal Dynamics of Chemoattractant Concentrations. Immunity. 2017;47: 862-874.e3. doi:10.1016/j.immuni.2017.10.020

182. Thon JN, Dykstra BJ, Beaulieu LM. Platelet bioreactor: accelerated evolution of design and manufacture. Platelets. 2017;28: 472–477. doi:10.1080/09537104.2016.1265922

183. Eyer K, Doineau RCL, Castrillon CE, Briseño-Roa L, Menrath V, Mottet G, et al. Single-cell deep phenotyping of IgG-secreting cells for high-resolution immune monitoring. Nat Biotechnol. 2017;35: 977–982. doi:10.1038/nbt.3964

184. Silva AM, Almeida MI, Teixeira JH, Maia AF, Calin GA, Barbosa MA, et al. Dendritic Cell-derived Extracellular Vesicles mediate Mesenchymal Stem/Stromal Cell recruitment. Sci Rep. 2017;7: 1667. doi:10.1038/s41598-017-01809-x

185. Burian E, Probst F, Palla B, Riedel C, Saller MM, Cornelsen M, et al. Effect of hypoxia on the proliferation of porcine bone marrow-derived mesenchymal stem cells and adipose-derived mesenchymal stem cells in 2- and 3-dimensional culture. J Cranio-Maxillofacial Surg. 2017;45: 414–419. doi:10.1016/j.jcms.2016.12.014

186. Tran R, Myers DR, Denning G, Shields JE, Lytle AM, Alrowais H, et al. Microfluidic Transduction Harnesses Mass Transport Principles to Enhance Gene Transfer Efficiency. Mol Ther. 2017;25: 2372–2382. doi:10.1016/j.ymthe.2017.07.002

187. Zheng GXY, Terry JM, Belgrader P, Ryvkin P, Bent ZW, Wilson R, et al. Massively parallel digital transcriptional profiling of single cells. Nat Commun. 2017;8. doi:10.1038/ncomms14049

188. Du X, Huang F, Zhang S, Yao Y, Chen Y, Chen Y, et al. Carboxymethylcellulose with phenolic hydroxyl microcapsules enclosinggene-modified BMSCs for controlled BMP-2 release in vitro. Artif Cells, Nanomedicine Biotechnol. 2017;45: 1710–1720. doi:10.1080/21691401.2017.1282499

189. Ma Y, Han X, Quintana Bustamante O, Bessa De Castro R, Zhang K, Zhang P, et al. Highly efficient genome editing of human hematopoietic stem cells via a nano-silicon-blade delivery approach. Integr Biol (United Kingdom). 2017;9: 548–554. doi:10.1039/c7ib00060j

190. Islam M, Brink H, Blanche S, DiPrete C, Bongiorno T, Stone N, et al. Microfluidic Sorting of Cells by Viability Based on Differences in Cell Stiffness. Sci Rep. 2017;7: 1997. doi:10.1038/s41598-017-01807-z

191. Piccirilli M, Delfinis CP, Santoro A, Salvati M. Mesenchymal stem cells in lumbar spine surgery: A single institution experience about red bone marrow and fat tissue derived MSCs. J Neurosurg Sci. 2017;61: 124–133. doi:10.23736/S0390-5616.16.03266-X

192. Titmarsh DM, Tan CLL, Glass NR, Nurcombe V, Cooper-White JJ, Cool SM. Microfluidic screening reveals heparan sulfate enhances human mesenchymal stem cell growth by modulating fibroblast growth factor-2 transport. Stem Cells Transl Med. 2017;6: 1178–1190. doi:10.1002/sctm.16-0343

193. Thomsen AR, Aldrian C, Bronsert P, Thomann Y, Nanko N, Melin N, et al. A deep conical agarose microwell array for adhesion independent three-dimensional cell culture and dynamic volume measurement. Lab Chip. 2017;18: 179–189. doi:10.1039/c7lc00832e

194. de la Rosa X, Norris PC, Chiang N, Rodriguez AR, Spur BW, Serhan CN. Identification and Complete Stereochemical Assignments of the New Resolvin Conjugates in Tissue Regeneration in Human Tissues that Stimulate Proresolving Phagocyte Functions and Tissue Regeneration. Am J Pathol. 2018;188: 950–966. doi:10.1016/j.ajpath.2018.01.004

195. Kamande JW, Lindell MAM, Witek MA, Voorhees PM, Soper SA. Isolation of circulating plasma cells from blood of patients diagnosed with clonal plasma cell disorders using cell selection microfluidics. Integr Biol (United Kingdom). 2018;10: 82–91. doi:10.1039/c7ib00183e

196. Jinnah AH, Zacks BC, Gwam CU, Kerr BA. Emerging and established models of bone metastasis. Cancers (Basel). 2018;10. doi:10.3390/cancers10060176

197. Holmes HL, Wilson B, Goerger JP, Silverberg JL, Cohen I, Zipfel WR, et al. Facilitated recruitment of mesenchymal stromal cells by bone marrow concentrate and platelet rich plasma. PLoS One. 2018;13: e0194567. doi:10.1371/journal.pone.0194567

198. Kinz E, Muendlein A. Quantitation of JAK2 V617F allele burden by using the quantstudioTM 3d digital PCR system. Methods Mol Biol. 2018;1768: 257–273. doi:10.1007/978-1-4939-7778-9_15

199. Schneider E, Staffas A, Röhner L, Malmberg ED, Ashouri A, Krowiorz K, et al. Micro-ribonucleic acid-155 is a direct target of meis1, but not a driver in acute myeloid leukemia. Haematologica. 2018;103: 246–255. doi:10.3324/haematol.2017.177485

200. Liao CY, Song MJ, Gao Y, Mauer AS, Revzin A, Malhi H. Hepatocyte-Derived Lipotoxic Extracellular Vesicle Sphingosine 1-Phosphate Induces Macrophage Chemotaxis. Front Immunol. 2018;9: 2980. doi:10.3389/fimmu.2018.02980

201. Occhetta P, Pigeot S, Rasponi M, Dasen B, Mehrkens A, Ullrich T, et al. Developmentally inspired programming of adult human mesenchymal stromal cells toward stable chondrogenesis. Proc Natl Acad Sci U S A. 2018;115: 4625–4630. doi:10.1073/pnas.1720658115

202. Geraili A, Jafari P, Hassani MS, Araghi BH, Mohammadi MH, Ghafari AM, et al. Controlling Differentiation of Stem Cells for Developing Personalized Organ-on-Chip Platforms. Adv Healthc Mater. 2018;7: 1–26. doi:10.1002/adhm.201700426

203. Jenkins RW, Aref AR, Lizotte PH, Ivanova E, Stinson S, Zhou CW, et al. Ex vivo profiling of PD-1 blockade using organotypic tumor spheroids. Cancer Discov. 2018;8: 196–215. doi:10.1158/2159-8290.CD-17-0833

204. Leferink AM, Reis DS, Van Blitterswijk CA, Moroni L. An antibody based approach for multi-coloring osteogenic and chondrogenic proteins in tissue engineered constructs. Biomed Mater. 2018;13: 44102. doi:10.1088/1748-605X/aab51e

205. Winkler JW, Libreros S, De La Rosa X, Sansbury BE, Norris PC, Chiang N, et al. Frontline Science: Structural insights into Resolvin D4 actions and further metabolites via a new total organic synthesis and validation. J Leukoc Biol. 2018;103: 995–1010. doi:10.1002/JLB.3MI0617-254R

206. AbuZineh K, Joudeh LI, Al Alwan B, Hamdan SM, Merzaban JS, Habuchi S. Microfluidics-based super-resolution microscopy enables nanoscopic characterization of blood stem cell rolling. Sci Adv. 2018;4. doi:10.1126/sciadv.aat5304

207. Chen Y, Mao P, Snijders AM, Wang D. Senescence chips for ultrahigh-throughput isolation and removal of senescent cells. Aging Cell. 2018;17. doi:10.1111/acel.12722

208. Zeng Y, Gao L, Luo X, Chen Y, Kabeer MH, Chen X, et al. Microfluidic enrichment of plasma cells improves treatment of multiple myeloma. Mol Oncol. 2018;12: 1004–1011. doi:10.1002/1878-0261.12201

209. Moore LR, Mizutani D, Tanaka T, Buck A, Yazer M, Zborowski M, et al. Continuous, intrinsic magnetic depletion of erythrocytes from whole blood with a quadrupole magnet and annular flow channel; pilot scale study. Biotechnol Bioeng. 2018;115: 1521–1530. doi:10.1002/bit.26581

210. Hassoun M, Köse N, Kiselev R, Kirchberger-Tolstik T, Schie IW, Krafft C, et al. Quantitation of acute monocytic leukemia cells spiked in control monocytes using surface-enhanced Raman spectroscopy. Anal Methods. 2018;10: 2785–2791. doi:10.1039/c8ay01046c

211. Petukhov V, Guo J, Baryawno N, Severe N, Scadden DT, Samsonova MG, et al. dropEst: Pipeline for accurate estimation of molecular counts in droplet-based single-cell RNA-seq experiments. Genome Biol. 2018;19. doi:10.1186/s13059-018-1449-6

212. Zhang P, Samuel G, Crow J, Godwin AK, Zeng Y. Molecular assessment of circulating exosomes toward liquid biopsy diagnosis of Ewing sarcoma family of tumors. Transl Res. 2018;201: 136–153. doi:10.1016/j.trsl.2018.05.007

213. Hou Y, Xie W, Achazi K, Cuellar-Camacho JL, Melzig MF, Chen W, et al. Injectable degradable PVA microgels prepared by microfluidic technology for controlled osteogenic differentiation of mesenchymal stem cells. Acta Biomater. 2018;77: 28–37. doi:10.1016/j.actbio.2018.07.003

214. Rosendahl P, Plak K, Jacobi A, Kraeter M, Toepfner N, Otto O, et al. Real-time fluorescence and deformability cytometry. Nat Methods. 2018;15: 355–358. doi:10.1038/nmeth.4639

215. Dejima H, Nakanishi H, Kuroda H, Yoshimura M, Sakakura N, Ueda N, et al. Detection of abundant megakaryocytes in pulmonary artery blood in lung cancer patients using a microfluidic platform. Lung Cancer. 2018;125: 128–135. doi:10.1016/j.lungcan.2018.09.011

216. Lembong J, Lerman MJ, Kingsbury TJ, Civin CI, Fisher JP. A fluidic culture platform for spatially patterned cell growth, differentiation, and cocultures. Tissue Eng - Part A. 2018;24: 1715–1732. doi:10.1089/ten.tea.2018.0020

217. Hümmer J, Koc J, Rosenhahn A, Lee-Thedieck C. Microfluidic Shear Force Assay to Determine Cell Adhesion Forces. Methods Mol Biol. 2019;2017: 71–84. doi:10.1007/978-1-4939-9574-5_6

218. La Manno G. From single-cell RNA-seq to transcriptional regulation. Nat Biotechnol. 2019;37: 1421–1422. doi:10.1038/s41587-019-0327-4

219. Gold K, Gaharwar AK, Jain A. Emerging trends in multiscale modeling of vascular pathophysiology: Organ-on-a-chip and 3D printing. Biomaterials. 2019;196: 2–17. doi:10.1016/j.biomaterials.2018.07.029

220. Xavier M, Holm SH, Beech JP, Spencer D, Tegenfeldt JO, Oreffo ROC, et al. Label-free enrichment of primary human skeletal progenitor cells using deterministic lateral displacement. Lab Chip. 2019;19: 513–523. doi:10.1039/c8lc01154k

221. Park W, Jang S, Kim TW, Bae J, Oh TI, Lee EA. Microfluidic-Printed Microcarrier for In Vitro Expansion of Adherent Stem Cells in 3D Culture Platform. Macromol Biosci. 2019;19: e1900136. doi:10.1002/mabi.201900136

222. Li H, Kaiser TK, Borschiwer M, Bohnenberger H, Reichardt SD, Lühder F, et al. Glucocorticoid resistance of allogeneic T cells alters the gene expression profile in the inflamed small intestine of mice suffering from acute graft-versus-host disease. J Steroid Biochem Mol Biol. 2019;195: 105485. doi:10.1016/j.jsbmb.2019.105485

223. Lou G, Anderluzzi G, Woods S, Roberts CW, Perrie Y. A novel microfluidic-based approach to formulate size-tuneable large unilamellar cationic liposomes: Formulation, cellular uptake and biodistribution investigations. Eur J Pharm Biopharm. 2019;143: 51–60. doi:10.1016/j.ejpb.2019.08.013

224. Khoo BL, Shang M, Ng CH, Lim CT, Chng WJ, Han J. Liquid biopsy for minimal residual disease detection in leukemia using a portable blast cell biochip. npj Precis Oncol. 2019;3: 30. doi:10.1038/s41698-019-0102-5

225. Tien J. Tissue engineering of the microvasculature. Compr Physiol. 2019;9: 1155–1212. doi:10.1002/cphy.c180037

226. Um E, Oh JM, Park J, Song T, Kim TE, Choi Y, et al. Immature dendritic cells navigate microscopic mazes to find tumor cells. Lab Chip. 2019;19: 1665–1675. doi:10.1039/c9lc00150f

227. Du M, Kavanagh D, Zhang Z, Kalia N. Designing Microfluidic Devices to Sort Haematopoietic Stem Cells Based on Their Mechanical Properties. Stem Cells Int. 2019;2019. doi:10.1155/2019/8540706

228. Aljaghtham MS, Liu ZL, Guo JJ, He J, Celik E. Numerical simulations of cell flow and trapping within microfluidic channels for stiffness based cell isolation. J Biomech. 2019;85: 43–49. doi:10.1016/j.jbiomech.2019.01.010

229. Derakhshani M, Abbaszadeh H, Movassaghpour AA, Mehdizadeh A, Ebrahimi-Warkiani M, Yousefi M. Strategies for elevating hematopoietic stem cells expansion and engraftment capacity. Life Sci. 2019;232. doi:10.1016/j.lfs.2019.116598

230. Zhang Y, Fu X, Guo W, Deng Y, Binks BP, Shum HC. Electrocoalescence of liquid marbles driven by embedded electrodes for triggering bioreactions. Lab Chip. 2019;19: 3526–3534. doi:10.1039/c9lc00722a

231. Hagen MW, Hinds MT. Static spatial growth restriction micropatterning of endothelial colony forming cells influences their morphology and gene expression. PLoS One. 2019;14. doi:10.1371/journal.pone.0218197

232. Soroush F, Tang Y, Guglielmo K, Engelmann A, Liverani E, Patel A, et al. Protein Kinase C-Delta (PKCδ) Tyrosine Phosphorylation is a Critical Regulator of Neutrophil-Endothelial Cell Interaction in Inflammation. Shock. 2019;51: 538–547. doi:10.1097/SHK.0000000000001247

233. Leferink AM, Tibbe MP, Bossink EGBM, de Heus LE, van Vossen H, van den Berg A, et al. Shape-defined solid micro-objects from poly(D,L-lactic acid) as cell-supportive counterparts in bottom-up tissue engineering. Mater Today Bio. 2019;4: 100025. doi:10.1016/j.mtbio.2019.100025

234. Zhang C, Tu HL, Jia G, Mukhtar T, Taylor V, Rzhetsky A, et al. Ultra-multiplexed analysis of single-cell dynamics reveals logic rules in differentiation. Sci Adv. 2019;5. doi:10.1126/sciadv.aav7959_rfseq1

235. Shih YV, Varghese S. Tissue engineered bone mimetics to study bone disorders ex vivo: Role of bioinspired materials. Biomaterials. 2019;198: 107–121. doi:10.1016/j.biomaterials.2018.06.005

236. Ozbolat V, Dey M, Ayan B, Ozbolat IT. Extrusion-based printing of sacrificial Carbopol ink for fabrication of microfluidic devices. Biofabrication. 2019;11. doi:10.1088/1758-5090/ab10ae

237. Mandl HK, Quijano E, Suh HW, Sparago E, Oeck S, Grun M, et al. Optimizing biodegradable nanoparticle size for tissue-specific delivery. J Control Release. 2019;314: 92–101. doi:10.1016/j.jconrel.2019.09.020

238. Ouyang D, Li Y, He W, Lin W, Hu L, Wang C, et al. Mechanical segregation and capturing of clonal circulating plasma cells in multiple myeloma using micropillar-integrated microfluidic device. Biomicrofluidics. 2019;13: 64114. doi:10.1063/1.5112050

239. Liu S, Stroncek DF, Zhao Y, Chen V, Shi R, Chen J, et al. Single cell sequencing reveals gene expression signatures associated with bone marrow stromal cell subpopulations and time in culture. J Transl Med. 2019;17: 23. doi:10.1186/s12967-018-1766-2

240. Jahangiri M, Khosravi S, Moghtaderi H, Ranjbar M, Abadijoo H, Sarmadi S, et al. Microfluidic platform with integrated electrical actuator to enrich and locating atypical/cancer cells from liquid cytology samples. Sensors Actuators, B Chem. 2019;297: 126733. doi:10.1016/j.snb.2019.126733

241. Moloudi R, Oh S, Yang C, Teo KL, Lam ATL, Ebrahimi Warkiani M, et al. Scaled-Up Inertial Microfluidics: Retention System for Microcarrier-Based Suspension Cultures. Biotechnol J. 2019;14: e1800674. doi:10.1002/biot.201800674

242. Hu M, Liu P, Liu Y, Yue M, Wang Y, Wang S, et al. Platelet Shp2 negatively regulates thrombus stability under high shear stress. J Thromb Haemost. 2019;17: 220–231. doi:10.1111/jth.14335

243. Rashidian M, LaFleur MW, Verschoor VL, Dongre A, Zhang Y, Nguyen TH, et al. Immuno-PET identifies the myeloid compartment as a key contributor to the outcome of the antitumor response under PD-1 blockade. Proc Natl Acad Sci U S A. 2019;116: 16971–16980. doi:10.1073/pnas.1905005116

244. Xu L, Durruthy-Durruthy R, Eastburn DJ, Pellegrino M, Shah O, Meyer E, et al. Clonal Evolution and Changes in Two AML Patients Detected with A Novel Single-Cell DNA Sequencing Platform. Sci Rep. 2019;9: 11119. doi:10.1038/s41598-019-47297-z

245. Lareau CA, Duarte FM, Chew JG, Kartha VK, Burkett ZD, Kohlway AS, et al. Droplet-based combinatorial indexing for massive-scale single-cell chromatin accessibility. Nat Biotechnol. 2019;37: 916–924. doi:10.1038/s41587-019-0147-6

246. Witte K, Rodrigo-Navarro A, Salmeron-Sanchez M. Bacteria-laden microgels as autonomous three-dimensional environments for stem cell engineering. Mater Today Bio. 2019;2: 100011. doi:10.1016/j.mtbio.2019.100011

247. Thomas RSW, Mitchell PD, Oreffo ROC, Morgan H, Green NG. Image-based sorting and negative dielectrophoresis for high purity cell and particle separation. Electrophoresis. 2019;40: 2718–2727. doi:10.1002/elps.201800489

248. Moura PL, Iragorri MAL, Français O, Le Pioufle B, Dobbe JGG, Streekstra GJ, et al. Reticulocyte and red blood cell deformation triggers specific phosphorylation events. Blood Adv. 2019;3: 2653–2663. doi:10.1182/bloodadvances.2019000545

249. Jyotsana N, Sharma A, Chaturvedi A, Budida R, Scherr M, Kuchenbauer F, et al. Lipid nanoparticle-mediated siRNA delivery for safe targeting of human CML in vivo. Ann Hematol. 2019;98: 1905–1918. doi:10.1007/s00277-019-03713-y

250. Crow J, Samuel G, Godwin AK. Beyond tumor mutational burden: potential and limitations in using exosomes to predict response to immunotherapy. Expert Rev Mol Diagn. 2019;19: 1079–1088. doi:10.1080/14737159.2020.1688144

251. Mao AS, Özkale B, Shah NJ, Vining KH, Descombes T, Zhang L, et al. Programmable microencapsulation for enhanced mesenchymal stem cell persistence and immunomodulation. Proc Natl Acad Sci U S A. 2019;116: 15392–15397. doi:10.1073/pnas.1819415116

252. Kshitiz, Ellison DD, Suhail Y, Afzal J, Woo L, Kilic O, et al. Dynamic secretome of bone marrow-derived stromal cells reveals a cardioprotective biochemical cocktail. Proc Natl Acad Sci U S A. 2019;116: 14374–14383. doi:10.1073/pnas.1902598116

253. Fan W, Chen X, Ge Y, Jin Y, Jin Q, Zhao J. Single-cell impedance analysis of osteogenic differentiation by droplet-based microfluidics. Biosens Bioelectron. 2019;145: 111730. doi:10.1016/j.bios.2019.111730

254. Thibault D, Jensen PA, Wood S, Qabar C, Clark S, Shainheit MG, et al. Droplet Tn-Seq combines microfluidics with Tn-Seq for identifying complex single-cell phenotypes. Nat Commun. 2019;10. doi:10.1038/s41467-019-13719-9

255. [Anonymous]. 46th ESAO Congress 3–7 September 2019 Hannover, Germany. Int J Artif Organs. 2019;42: 386–474. doi:10.1177/0391398819860985

256. Whittam AJ, Maan ZN, Duscher D, Barrera JA, Hu MS, Fischer LH, et al. Small molecule inhibition of dipeptidyl peptidase-4 enhances bone marrow progenitor cell function and angiogenesis in diabetic wounds. Transl Res. 2019;205: 51–63. doi:10.1016/j.trsl.2018.10.006

257. Rinoldi C, Costantini M, Kijeńska-Gawrońska E, Testa S, Fornetti E, Heljak M, et al. Tendon Tissue Engineering: Effects of Mechanical and Biochemical Stimulation on Stem Cell Alignment on Cell-Laden Hydrogel Yarns. Adv Healthc Mater. 2019;8. doi:10.1002/adhm.201801218

258. Han X, Ma Y, Zhang K, Zhang P, Shao N, Qin L. Microfluidic Cell Trap Arrays for Single Hematopoietic Stem/Progenitor Cell Behavior Analysis. Proteomics. 2019;20: e1900223. doi:10.1002/pmic.201900223

259. Du M, Kavanagh D, Kalia N, Zhang Z. Characterising the mechanical properties of haematopoietic and mesenchymal stem cells using micromanipulation and atomic force microscopy. Med Eng Phys. 2019;73: 18–29. doi:10.1016/j.medengphy.2019.07.013

260. Moore N, Chevillet JR, Healey LJ, McBrine C, Doty D, Santos J, et al. A Microfluidic Device to Enhance Viral Transduction Efficiency During Manufacture of Engineered Cellular Therapies. Sci Rep. 2019;9: 15101. doi:10.1038/s41598-019-50981-9

261. Crookenden MA, Phyn CVC, Turner SA, Loor JJ, Smith AI, Lopreiato V, et al. Feeding synthetic zeolite to transition dairy cows alters neutrophil gene expression. J Dairy Sci. 2020;103: 723–736. doi:10.3168/jds.2019-17097

262. Feng Q, Gao H, Wen H, Huang H, Li Q, Liang M, et al. Engineering the cellular mechanical microenvironment to regulate stem cell chondrogenesis: Insights from a microgel model. Acta Biomater. 2020. doi:10.1016/j.actbio.2020.06.046

263. Yang J, Zhu Y, Wang F, Deng L, Xu X, Cui W. Microfluidic liposomes-anchored microgels as extended delivery platform for treatment of osteoarthritis. Chem Eng J. 2020;400: 126004. doi:10.1016/j.cej.2020.126004

264. Sart S, Tomasi RFX, Barizien A, Amselem G, Cumano A, Baroud CN. Mapping the structure and biological functions within mesenchymal bodies using microfluidics. Sci Adv. 2020;6. doi:10.1126/sciadv.aaw7853

265. Wu J, Li G, Ye T, Lu G, Li R, Deng L, et al. Stem cell-laden injectable hydrogel microspheres for cancellous bone regeneration. Chem Eng J. 2020;393: 124715. doi:10.1016/j.cej.2020.124715

266. Chen Z, Jiang K, Zou Z, Luo X, Lim CT, Wen C. High-throughput and label-free isolation of senescent murine mesenchymal stem cells. Biomicrofluidics. 2020;14: 034106. doi:10.1063/5.0011925

267. Lindemann O, Rossaint J, Najder K, Schimmelpfennig S, Hofschröer V, Wälte M, et al. Intravascular adhesion and recruitment of neutrophils in response to CXCL1 depends on their TRPC6 channels. J Mol Med. 2020;98: 349–360. doi:10.1007/s00109-020-01872-4

268. Grigolato F, Egholm C, Impellizzieri D, Arosio P, Boyman O. Establishment of a scalable microfluidic assay for characterization of population-based neutrophil chemotaxis. Allergy Eur J Allergy Clin Immunol. 2020;75: 1382–1393. doi:10.1111/all.14195

269. Maharjan S, Cecen B, Zhang YS. 3D Immunocompetent Organ-on-a-Chip Models. Small Methods. 2020. doi:10.1002/smtd.202000235

270. Ribeiro P, Leitão L, Monteiro AC, Bortolin A, Moura B, Lamghari M, et al. Microfluidic-based models to address the bone marrow metastatic niche complexity. Semin Cell Dev Biol. 2020. doi:10.1016/j.semcdb.2020.05.015

271. Lu M, Rab MAE, Shevkoplyas SS, Sheehan VA. Blood rheology biomarkers in sickle cell disease. Exp Biol Med. 2020;245: 155–165. doi:10.1177/1535370219900494

272. Rambøl MH, Han E, Niklason LE. Microvessel Network Formation and Interactions with Pancreatic Islets in Three-Dimensional Chip Cultures. Tissue Eng - Part A. 2020;26: 556–568. doi:10.1089/ten.tea.2019.0186

273. Belling JN, Heidenreich LK, Tian Z, Mendoza AM, Chiou TT, Gong Y, et al. Acoustofluidic sonoporation for gene delivery to human hematopoietic stem and progenitor cells. Proc Natl Acad Sci U S A. 2020;117: 10976–10982. doi:10.1073/pnas.1917125117

274. Singh NK, Han W, Nam SA, Kim JW, Kim JY, Kim YK, et al. Three-dimensional cell-printing of advanced renal tubular tissue analogue. Biomaterials. 2020;232. doi:10.1016/j.biomaterials.2019.119734

275. Liu D, Paczkowski P, Mackay S, Ng C, Zhou J. Single-Cell Multiplexed Proteomics on the IsoLight Resolves Cellular Functional Heterogeneity to Reveal Clinical Responses of Cancer Patients to Immunotherapies. Methods Mol Biol. 2020;2055: 413–431. doi:10.1007/978-1-4939-9773-2_19

276. Jin L, Zhao W, Zhang J, Chen W, Xie T, Wang L, et al. Evaluation of the diagnostic value of circulating tumor cells with CytoSorter® CTC capture system in patients with breast cancer. Cancer Med. 2020;9: 1638–1647. doi:10.1002/cam4.2825

277. İçöz K, Akar Ü, Ünal E. Microfluidic Chip based direct triple antibody immunoassay for monitoring patient comparative response to leukemia treatment. Biomed Microdevices. 2020;22: 48. doi:10.1007/s10544-020-00503-6

278. Pagella P, Miran S, Neto E, Martin I, Lamghari M, Mitsiadis TA. Human dental pulp stem cells exhibit enhanced properties in comparison to human bone marrow stem cells on neurites outgrowth. FASEB J. 2020;34: 5499–5511. doi:10.1096/fj.201902482R

279. M. Weerakoon-Ratnayake K, Vaidyanathan S, Larkey N, Dathathreya K, Hu M, Jose J, et al. Microfluidic Device for On-Chip Immunophenotyping and Cytogenetic Analysis of Rare Biological Cells. Cells. 2020;9: 519. doi:10.3390/cells9020519

280. Malek-Khatabi A, Javar HA, Dashtimoghadam E, Ansari S, Hasani-Sadrabadi MM, Moshaverinia A. In situ bone tissue engineering using gene delivery nanocomplexes. Acta Biomater. 2020;108: 326–336. doi:10.1016/j.actbio.2020.03.008

**Second step** –The introduction, results, discussion, and conclusions of the selected publications were systematically screened. Publications that did not mimic at least one BM feature (e.g., specific niches, cell niche interactions, or BM microenvironment) or that assessed only isolated BM phenomena, without associating them with the structural aspects of the BM were excluded (poor mimicking):

1. Faley SL, Copland M, Wlodkowic D, Kolch W, Seale KT, Wikswo JP, et al. Microfluidic single cell arrays to interrogate signalling dynamics of individual, patient-derived hematopoietic stem cellsY. Lab Chip. 2009;9: 2659–2664. doi:10.1039/b902083g

2. Sonntag F, Schilling N, Mader K, Gruchow M, Klotzbach U, Lindner G, et al. Design and prototyping of a chip-based multi-micro-organoid culture system for substance testing, predictive to human (substance) exposure. J Biotechnol. 2010;148: 70–75. doi:10.1016/j.jbiotec.2010.02.001

3. Trkov S, Eng G, Di Liddo R, Parnigotto PP, Vunjak-Novakovic G. Micropatterned three-dimensional hydrogel system to study human endothelial-mesenchymal stem cell interactions. J Tissue Eng Regen Med. 2010;4: 205–215. doi:10.1002/term.231

4. Lecault V, Vaninsberghe M, Sekulovic S, Knapp DJHF, Wohrer S, Bowden W, et al. High-throughput analysis of single hematopoietic stem cell proliferation in microfluidic cell culture arrays. Nat Methods. 2011;8: 581–589. doi:10.1038/nmeth.1614

5. Hansmann G, Plouffe BD, Hatch A, Von Gise A, Sallmon H, Zamanian RT, et al. Design and validation of an endothelial progenitor cell capture chip and its application in patients with pulmonary arterial hypertension. J Mol Med. 2011;89: 971–983. doi:10.1007/s00109-011-0779-6

6. Wu HW, Lin CC, Hwang SM, Chang YJ, Lee G Bin. A microfluidic device for chemical and mechanical stimulation of mesenchymal stem cells. Microfluid Nanofluidics. 2011;11: 545–556. doi:10.1007/s10404-011-0820-7

7. Liu Z, Xiao L, Xu B, Zhang Y, Mak AFT, Li Y, et al. Covalently immobilized biomolecule gradient on hydrogel surface using a gradient generating microfluidic device for a quantitative mesenchymal stem cell study. Biomicrofluidics. 2012;6. doi:10.1063/1.4704522

8. Tian X, Wang S, Zhang Z, Lv D. Rat bone marrow-derived Schwann-like cells differentiated by the optimal inducers combination on microfluidic chip and their functional performance. PLoS One. 2012;7: 1–11. doi:10.1371/journal.pone.0042804

9. Park SH, Sim WY, Min BH, Yang SS, Khademhosseini A, Kaplan DL. Chip-Based Comparison of the Osteogenesis of Human Bone Marrow- and Adipose Tissue-Derived Mesenchymal Stem Cells under Mechanical Stimulation. PLoS One. 2012;7: 1–11. doi:10.1371/journal.pone.0046689

10. Menon N V., Chuah YJ, Cao B, Lim M, Kang Y. A microfluidic co-culture system to monitor tumor-stromal interactions on a chip. Biomicrofluidics. 2014;8. doi:10.1063/1.4903762

11. Sun Y, Yao Z, Lin P, Hou X, Chen L. Bone marrow mesenchymal stem cells ameliorate inflammatory factor-induced dysfunction of INS-1 cells on chip. Cell Biol Int. 2014;38: 647–654. doi:10.1002/cbin.10248

12. Gao X, Zhang X, Xu H, Zhou B, Wen W, Qin J. Regulation of cell migration and osteogenic differentiation in mesenchymal stem cells under extremely low fluidic shear stress. Biomicrofluidics. 2014;8. doi:10.1063/1.4896557

13. Rexius-Hall ML, Mauleon G, Malik AB, Rehman J, Eddington DT. Microfluidic platform generates oxygen landscapes for localized hypoxic activation. Lab Chip. 2014;14: 4688–4695. doi:10.1039/c4lc01168f

14. Jeon JS, Bersini S, Whisler JA, Chen MB, Dubini G, Charest JL, et al. Generation of 3D functional microvascular networks with human mesenchymal stem cells in microfluidic systems. Integr Biol (United Kingdom). 2014;6: 555–563. doi:10.1039/c3ib40267c

15. Dixon AR, Rajan S, Kuo CH, Bersano T, Wold R, Futai N, et al. Microfluidic device capable of medium recirculation for non-adherent cell culture. Biomicrofluidics. 2014;8. doi:10.1063/1.4865855

16. Bersini S, Jeon JS, Dubini G, Arrigoni C, Chung S, Charest JL, et al. A microfluidic 3D invitro model for specificity of breast cancer metastasis to bone. Biomaterials. 2014;35: 2454–2461. doi:10.1016/j.biomaterials.2013.11.050

17. Lin H, Lozito TP, Alexander PG, Gottardi R, Tuan RS. Stem Cell-Based Microphysiological Osteochondral System to Model Tissue Response to Interleukin-1 beta. Mol Pharm. 2014;11: 2203–2212. doi:10.1021/mp500136b

18. Mahadik BP, Wheeler TD, Skertich LJ, Kenis PJA, Harley BAC. Microfluidic Generation of Gradient Hydrogels to Modulate Hematopoietic Stem Cell Culture Environment. Adv Healthc Mater. 2014;3: 449–458. doi:10.1002/adhm.201300263

19. Ahn B, Wang Z, Archer DR, Lam WA. Using microfluidics to investigate hematopoietic stem cell and microniche interactions at the single cell level. Methods Mol Biol. 2014;1185: 223–233. doi:10.1007/978-1-4939-1133-2_15

20. Hong S, Jung Y, Yen R, Chan HF, Leong KW, Truskey GA, et al. Magnetoactive sponges for dynamic control of microfluidic flow patterns in microphysiological systems. Lab Chip. 2014;14: 514–521. doi:10.1039/c3lc51076j

21. Tsao CW, Cheng YC, Cheng JH. Fluid flow shear stress stimulation on a multiplex microfluidic device for rat bone marrow stromal cell differentiation enhancement. Micromachines. 2015;6: 1996–2009. doi:10.3390/mi6121470

22. Poon Z, Lee WC, Guan G, Nyan LM, Lim CT, Han J, et al. Bone Marrow Regeneration Promoted by Biophysically Sorted Osteoprogenitors From Mesenchymal Stromal Cells. Stem Cells Transl Med. 2015;4: 56–65. doi:10.5966/sctm.2014-0154

23. Silva A, Jacobson T, Meads M, Distler A, Shain K. An organotypic high throughput system for characterization of drug sensitivity of primary multiple myeloma cells. J Vis Exp. 2015;2015: 1–12. doi:10.3791/53070

24. Mahadik BP, Pedron Haba S, Skertich LJ, Harley BAC. The use of covalently immobilized stem cell factor to selectively affect hematopoietic stem cell activity within a gelatin hydrogel. Biomaterials. 2015;67: 297–307. doi:10.1016/j.biomaterials.2015.07.042

25. Ding S, Li L, Liu X, Yang G, Zhou G, Zhou S. A nano-micro alternating multilayer scaffold loading with rBMSCs and BMP-2 for bone tissue engineering. Colloids Surfaces B Biointerfaces. 2015;133: 286–295. doi:10.1016/j.colsurfb.2015.06.015

26. Cambier T, Honegger T, Vanneaux V, Berthier J, Peyrade D, Blanchoin L, et al. Design of a 2D no-flow chamber to monitor hematopoietic stem cells. Lab Chip. 2015;15: 77–85. doi:10.1039/c4lc00807c

27. Occhetta P, Centola M, Tonnarelli B, Redaelli A, Martin I, Rasponi M. High-throughput microfluidic platform for 3D cultures of mesenchymal stem cells, towards engineering developmental processes. Sci Rep. 2015;5: 1–12. doi:10.1038/srep10288

28. Menon N V., Chuah YJ, Phey S, Zhang Y, Wu Y, Chan V, et al. Microfluidic Assay To Study the Combinatorial Impact of Substrate Properties on Mesenchymal Stem Cell Migration. ACS Appl Mater Interfaces. 2015;7: 17095–17103. doi:10.1021/acsami.5b03753

29. Chuah YJ, Koh YT, Lim K, Menon N V., Wu Y, Kang Y. Simple surface engineering of polydimethylsiloxane with polydopamine for stabilized mesenchymal stem cell adhesion and multipotency. Sci Rep. 2015;5: 1–12. doi:10.1038/srep18162

30. Campillo N, Jorba I, Schaedel L, Casals B, Gozal D, Farré R, et al. A novel chip for cyclic stretch and intermittent hypoxia cell exposures mimicking obstructive sleep apnea. Front Physiol. 2016;7: 319. doi:10.3389/fphys.2016.00319

31. Blin A, Le Goff A, Magniez A, Poirault-Chassac S, Teste B, Sicot G, et al. Microfluidic model of the platelet-generating organ: Beyond bone marrow biomimetics. Sci Rep. 2016;6: 1–12. doi:10.1038/srep21700

32. Schwarz J, Bierbaum V, Merrin J, Frank T, Hauschild R, Bollenbach T, et al. A microfluidic device for measuring cell migration towards substrate-bound and soluble chemokine gradients. Sci Rep. 2016;6: 36440. doi:10.1038/srep36440

33. Moore TA, Young EWK. Single cell functional analysis of multiple myeloma cell populations correlates with diffusion profiles in static microfluidic coculture systems. Biomicrofluidics. 2016;10. doi:10.1063/1.4958982

34. Portalska KJ, Chamberlain MD, Lo C, van Blitterswijk C, Sefton M V., de Boer J. Collagen modules for in situ delivery of mesenchymal stromal cell-derived endothelial cells for improved angiogenesis. J Tissue Eng Regen Med. 2016;10: 363–373. doi:10.1002/term.1738

35. Tatárová Z, Abbuehl JP, Maerkl S, Huelsken J. Microfluidic co-culture platform to quantify chemotaxis of primary stem cells. Lab Chip. 2016;16: 1934–1945. doi:10.1039/c6lc00236f

36. Khalil S, El-Badri N, El-Mokhtaar M, Al-Mofty S, Farghaly M, Ayman R, et al. A cost-effective method to assemble biomimetic 3D cell culture platforms. PLoS One. 2016;11. doi:10.1371/journal.pone.0167116

37. Yoon D, Kim H, Lee E, Park MH, Chung S, Jeon H, et al. Study on chemotaxis and chemokinesis of bone marrow-derived mesenchymal stem cells in hydrogel-based 3D microfluidic devices. Biomater Res. 2016;20: 1–8. doi:10.1186/s40824-016-0070-6

38. El-Badawy A, Amer M, Abdelbaset R, Sherif SN, Abo-Elela M, Ghallab YH, et al. Adipose stem cells display higher regenerative capacities and more adaptable electro-kinetic properties compared to bone marrow-derived mesenchymal stromal cells. Sci Rep. 2016;6: 37801. doi:10.1038/srep37801

39. Adeniran-Catlett AE, Beguin E, Bozal FK, Murthy SK. Suspension-based differentiation of adult mesenchymal stem cells toward chondrogenic lineage. Connect Tissue Res. 2016;57: 466–475. doi:10.3109/03008207.2015.1083989

40. Li F, Truong VX, Thissen H, Frith JE, Forsythe JS. Microfluidic Encapsulation of Human Mesenchymal Stem Cells for Articular Cartilage Tissue Regeneration. ACS Appl Mater Interfaces. 2017;9: 8589–8601. doi:10.1021/acsami.7b00728

41. Castellanos G, Nasim S, Almora DM, Rath S, Ramaswamy S. Stem Cell Cytoskeletal Responses to Pulsatile Flow in Heart Valve Tissue Engineering Studies. Front Cardiovasc Med. 2018;5: 58. doi:10.3389/fcvm.2018.00058

42. Dettinger P, Frank T, Etzrodt M, Ahmed N, Reimann A, Trenzinger C, et al. Automated Microfluidic System for Dynamic Stimulation and Tracking of Single Cells. Anal Chem. 2018;90: 10695–10700. doi:10.1021/acs.analchem.8b00312

43. Lee LM, Rosano JM, Wang Y, Klarmann GJ, Garson CJ, Prabhakarpandian B, et al. Label-free mesenchymal stem cell enrichment from bone marrow samples by inertial microfluidics. Anal Methods. 2018;10: 713–721. doi:10.1039/c7ay02500a

44. Vaez SA, Ebrahimi-Barough S, Soleimani M, Kolivand S, Farzamfar S, Tafti SHA, et al. The cardiac niche role in cardiomyocyte differentiation of rat bone marrow-derived stromal cells: Comparison between static and microfluidic cell culture methods. EXCLI J. 2018;17: 762–774. doi:10.17179/excli2018-1539

45. Ma HP, Deng X, Chen DY, Zhu D, Tong JL, Zhao T, et al. A microfluidic chip-based co-culture of fibroblast-like synoviocytes with osteoblasts and osteoclasts to test bone erosion and drug evaluation. R Soc Open Sci. 2018;5: 180528. doi:10.1098/rsos.180528

46. Hao S, Ha L, Cheng G, Wan Y, Xia Y, Sosnoski DM, et al. A Spontaneous 3D Bone-On-a-Chip for Bone Metastasis Study of Breast Cancer Cells. Small. 2018;14: e1702787. doi:10.1002/smll.201702787

47. Choudhary S, Sun Q, Mannion C, Kissin Y, Zilberberg J, Lee WY. Hypoxic Three-Dimensional Cellular Network Construction Replicates Ex Vivo the Phenotype of Primary Human Osteocytes. Tissue Eng - Part A. 2018;24: 458–468. doi:10.1089/ten.tea.2017.0103

48. Chen Z, He S, Zilberberg J, Lee W. Pumpless platform for high-throughput dynamic multicellular culture and chemosensitivity evaluation. Trans Annu Meet Soc Biomater Annu Int Biomater Symp. 2019;40: 705. doi:10.1039/C8LC00872H

49. Sarigil O, Anil-Inevi M, Yilmaz E, Mese G, Tekin HC, Ozcivici E. Label-free density-based detection of adipocytes of bone marrow origin using magnetic levitation. Analyst. 2019;144: 2942–2953. doi:10.1039/c8an02503g

50. Idaszek J, Costantini M, Karlsen TA, Jaroszewicz J, Colosi C, Testa S, et al. 3D bioprinting of hydrogel constructs with cell and material gradients for the regeneration of full-thickness chondral defect using a microfluidic printing head. Biofabrication. 2019;11: 44101. doi:10.1088/1758-5090/ab2622

51. Six KR, Sicot G, Devloo R, Feys HB, Baruch D, Compernolle V. A comparison of haematopoietic stem cells from umbilical cord blood and peripheral blood for platelet production in a microfluidic device. Vox Sang. 2019;114: 330–339. doi:10.1111/vox.12776

52. Liu Q, Li H, Lam KY. Modeling of a fast-response magnetic-sensitive hydrogel for dynamic control of microfluidic flow. Phys Chem Chem Phys. 2019;21: 1852–1862. doi:10.1039/c8cp06556j

53. Sharifi F, Patel BB, McNamara MC, Meis PJ, Roghair MN, Lu M, et al. Photo-Cross-Linked Poly(ethylene glycol) Diacrylate Hydrogels: Spherical Microparticles to Bow Tie-Shaped Microfibers. ACS Appl Mater Interfaces. 2019;11: 18797–18807. doi:10.1021/acsami.9b05555

54. Lundin V, Sugden WW, Theodore LN, Sousa PM, Han A, Chou S, et al. YAP Regulates Hematopoietic Stem Cell Formation in Response to the Biomechanical Forces of Blood Flow. Dev Cell. 2020;52: 446-460.e5. doi:10.1016/j.devcel.2020.01.006

55. Chuah YJ, Heng ZT, Tan JS, Tay LM, Lim CS, Kang Y, et al. Surface modifications to polydimethylsiloxane substrate for stabilizing prolonged bone marrow stromal cell culture. Colloids Surfaces B Biointerfaces. 2020;191: 110995. doi:10.1016/j.colsurfb.2020.110995

56. Lyu J, Chen L, Zhang J, Kang X, Wang Y, Wu W, et al. A microfluidics-derived growth factor gradient in a scaffold regulates stem cell activities for tendon-to-bone interface healing. Biomater Sci. 2020;8: 3649–3663. doi:10.1039/d0bm00229a

57. Witkowski MT, Dolgalev I, Evensen NA, Ma C, Chambers T, Roberts KG, et al. Extensive Remodeling of the Immune Microenvironment in B Cell Acute Lymphoblastic Leukemia. Cancer Cell. 2020;37: 867-882.e12. doi:10.1016/j.ccell.2020.04.015

58. Olm F, Lim HC, Schallmoser K, Strunk D, Laurell T, Scheding S. Acoustophoresis enables the label‐free separation of functionally different subsets of cultured bone marrow stromal cells. Cytom Part A. 2020. doi:10.1002/cyto.a.24171

59. Yin L, Yang Z, Wu Y, Denslin V, Yu CC, Tee CA, et al. Label-free separation of mesenchymal stem cell subpopulations with distinct differentiation potencies and paracrine effects. Biomaterials. 2020;240: 119881. doi:10.1016/j.biomaterials.2020.119881

60. Lin KC, Sun Y, Torga G, Sherpa P, Zhao Y, Qu J, et al. An in vitro tumor swamp model of heterogeneous cellular and chemotherapeutic landscapes. Lab Chip. 2020;20: 2453–2464. doi:10.1039/d0lc00131g

61. Carreras P, Gonzalez I, Gallardo M, Ortiz-Ruiz A, Martinez-Lopez J. Droplet microfluidics for the ex vivo expansion of human primary multiple myeloma cells. Micromachines. 2020;11. doi:10.3390/mi11030261

62. Sugimura R, Ohta R, Mori C, Li A, Mano T, Sano E, et al. Biomimetic aorta-gonad-Mesonephros-on-a-Chip to study human developmental hematopoiesis. Biomed Microdevices. 2020;22. doi:10.1007/s10544-020-00488-2
